# Variations on the Bergman Cyclization Theme: Electrocyclizations of Ionic Penta-, Hepta-, and Octadiynes

**DOI:** 10.1021/jacs.3c06691

**Published:** 2023-09-25

**Authors:** Dominic A. Sirianni, Xinli Song, Salmika Wairegi, Evan B. Wang, Sebastian A. Mendoza-Gomez, Adam Luxon, Maxwell Zimmerley, Ariana Nussdorf, Michael Filatov, Roald Hoffmann, Carol A. Parish

**Affiliations:** † Department of Natural Sciences, Daemen University, Amherst, New York 14226, United States; ‡ Department of Chemistry, University of Richmond, Richmond, Virginia 23173, United States; § Department of Chemistry, Southern Methodist University, Dallas, Texas 75275, United States; ∥ Department of Chemistry, Cornell University, Ithaca, New York 14853, United States

## Abstract

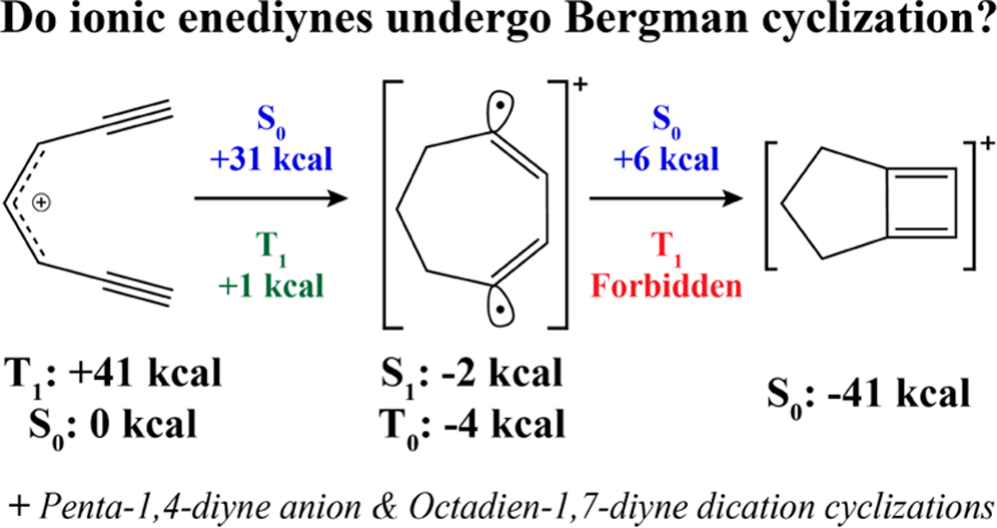

The Bergman cyclization of (*Z*)-hexa-3-ene-1,5-diyne to form the aromatic diradical *p*-benzyne has garnered attention as a potential antitumor agent due to its relatively low cyclization barrier and the stability of the resulting diradical. Here, we present a theoretical investigation of several ionic extensions of the fundamental Bergman cyclization: electrocyclizations of the penta-1,4-diyne anion, hepta-1,6-diyne cation, and octa-1,7-diyne dication, leveraging the spin-flip formulation of the equation-of-motion coupled cluster theory with single and double substitutions (EOM-SF-CCSD). Though the penta-1,4-diyne anion exhibits a large cyclization barrier of +66 kcal mol^–1^, cyclization of both the hepta-1,6-diyne cation and octa-1,7-diyne dication along a previously unreported triplet pathway requires relatively low energy. We also identified the presence of significant aromaticity in the triplet diradical products of these two cationic cyclizations.

## Introduction

**Scheme 1:**
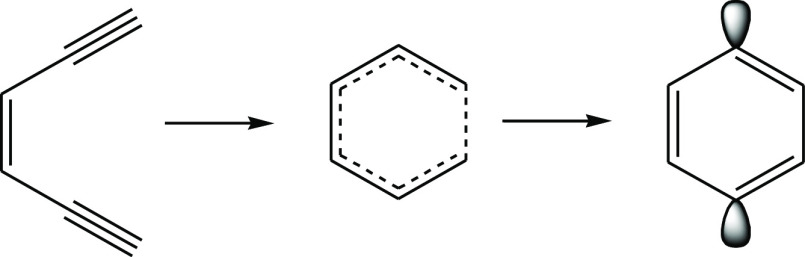
Bergman Cyclization of (*Z*)-Hexa-3-ene-1,5-diyne Forming Cyclic Diradical *p*-Benzyne

Bergman cyclization of (*Z*)-hexa-3-ene-1,5-diyne has been studied as a model for generating diradical *p*-benzyne-containing antitumor therapeutics.^1−4^ The DNA degradation activity is expected to occur upon excitation of the ground state diradical singlet to the decoupled triplet, which abstracts hydrogen atoms inducing cell death.^5^ This reaction has been well studied experimentally^6−11^ and computationally^12−35^ (Scheme 1). It is not surprising that the activation barrier (28.2 kcal mol^–1^) for this rearrangement is relatively small, as it is an interrupted, stepwise, symmetry-allowed [2 + 2] electrocyclization reaction.^17,36−40^ More remarkable is that its thermodynamic cost is only 8.5 kcal mol^–1^; the low endothermicity is the result of aromatization and the inherent thermodynamic instability of multiple bonds.^41^ The latter is exemplified by the strainless reaction of three ethylenes to form cyclohexane, which has been shown both experimentally and theoretically to be exothermic by nearly 70 kcal mol^–1^. Similarly, the reaction of four acetylenes to form cyclooctatetraene is exothermic by 146 kcal mol^–1^.^42^

**Scheme 2:**
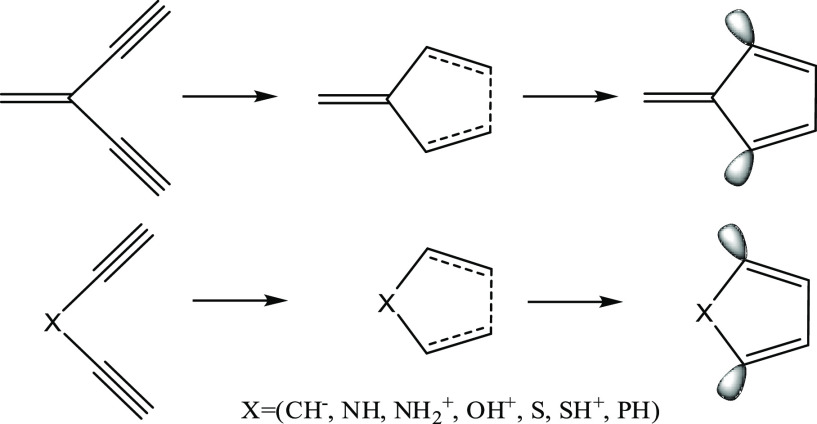
Extensions of the Bergman Cyclization to Functionalized 1,4-Pentadiynes by Kawatkar and Schreiner^43^ Reproduced from ref (43). Copyright 2002 American Chemical Society.

Kawatkar and Schreiner^43^ have shown that the aromatic driving force could be harnessed for the 1,5-cyclization of 3-substituted 1,4-pentadiynes. Because the radical orbitals are coplanar with the ring and are therefore unavailable for conjugation in the π system, the two “extra” electrons in this case are provided by a double bond at the 3-position or from a 3-substituent that contains lone pairs in conjugation with the π system (Scheme 2). Both the heteroatom and substituent effects were investigated in their study, which found that the ideal substituent X should be σ-withdrawing and π-donating, and the lowest cyclization barrier (for X=OH^+^) was computed to be +34.9 kcal mol^–1^ at the BLYP/6-31G* level of theory. Clearly, in order to experimentally harness the aromatic driving force for these cyclizations, enediynes must be designed with lower cyclization barriers and more favorable energies of reaction.

Aromaticity as a driving force was explored by Wu and co-workers. The gain of aromaticity and electronic complementarity has been shown to drive base pair selectivity.^44^ The same group has also shown that changes in aromaticity can affect hydrogen-bonding of π-conjugated heterocycles^45^ and influence the properties of annulated rings^46^ and other species.^47,48^

Bergman cyclizations of ionic species have not been fully explored either experimentally or computationally. Alabugin and co-workers have explored 1,5- and 1,6-cyclization in radical anions^12^ and harnessed aromaticity in driving cyclization in five-centered, six-electron anionic 5-endo species.^40^ Experimentally, the same group has reported an elegant and well-reasoned approach to Bergman cyclization of anionic enediynes by electron or hole injection.^49^

For the canonical Bergman cyclization, it has been suggested that bringing together the two acetylenic carbons to induce the formation of a new C–C bond contributes to a lowering of the activation energy.^2^ For cyclization to proceed, it has been proposed that the distance between terminal acetylenic carbons must fall within a “critical range” of approximately 2.9–3.4 Å, but no predictive relationship between this distance, the -yne orientation, and the activation energy has been demonstrated.^50−52^

**Scheme 3:**
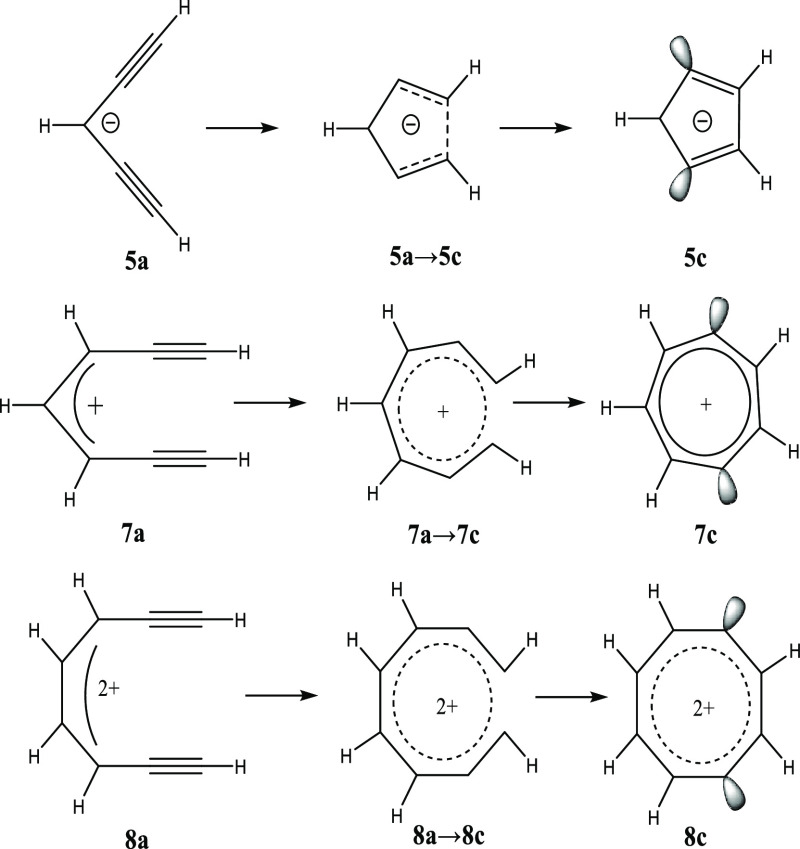
Ionic Variations on the Bergman Cyclization

**Scheme 4:**
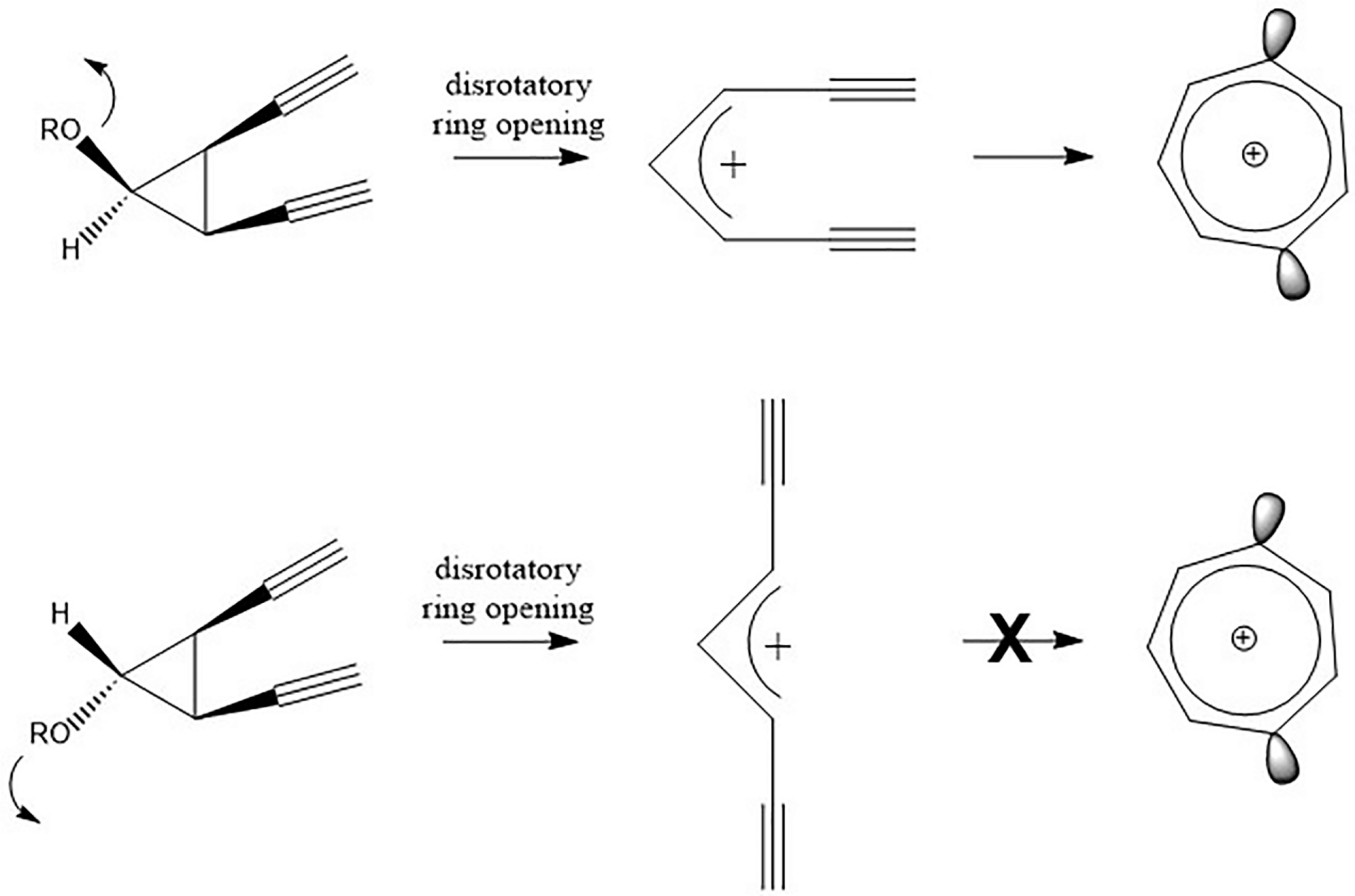
Generation of the Hepta-1,6-diyne Cation (**7a**) via the Disrotatory Ring Opening of a Cyclopropyl Precursor

In this work, we explore the aromatic driving force for ionic extensions of the Bergman cyclization (Scheme 3) and investigate the electronic structures and energetics of cyclization specifically for penta-1,4-diyn-3-ide (Scheme 3, top), (*Z*)-hepta-4-en-1,6-diyn-3-ylium (Scheme 3, middle), and octa-3,5-dien-1,7-diyne-4,5-diylium (Scheme 3, bottom). In the following, we will abbreviate these IUPAC names to **5a** → penta-1,4-diyne anion; **7a** → hepta-1,6-diyne cation, and **8a** → octa-1,7-diyne dication. To the best of our knowledge, no theoretical studies of the cationic Bergman-type cyclization have been reported previously, and these results yield interesting insights into the bonding of aromatic diradicals. Furthermore, the cyclization of the hepta-1,6-diyne cation is of particular interest for possible drug design because (in principle) it could be selectively obtained from the disrotatory ring-opening of a cyclopropyl precursor containing a stereospecifically placed leaving group, as shown in Scheme 4.

## Results and Discussion

We performed geometry optimizations of all reactants, transition states, and products in the cyclizations shown in Scheme 3 using the spin-flip (SF) formulation of equation-of-motion coupled cluster theory with single and double substitutions (EOM-SF-CCSD)^53−62^ along with the correlation-consistent polarized-valence double-ζ (cc-pVDZ) basis set. The SF approach allows for an accurate characterization of multiconfigurational systems by optimizing the single-reference triplet state and then performing spin flip transitions to determine the energy and orbital occupations of the resulting singlet states (see Section S1A in the Supporting Information for additional details). By fully characterizing the energetic profile of each Bergman-type cyclization presented in Scheme 3, we determined that the cyclization of the penta-1,4-diyne anion (Scheme 3, top) has a significant cyclization barrier of +66 kcal mol^–1^, which is thermally inaccessible under physiological conditions. We will therefore restrict our discussion to the cyclizations of the hepta-1,6-diyne cation and octa-1,7-diyne dication (Scheme 3, middle and bottom, respectively); for a full analysis of the cyclization of the penta-1,4-diyne anion, refer to Section SIIA in the Supporting Information.

### Cyclization of the Hepta-1,6-diyne Cation

**Figure 1:**
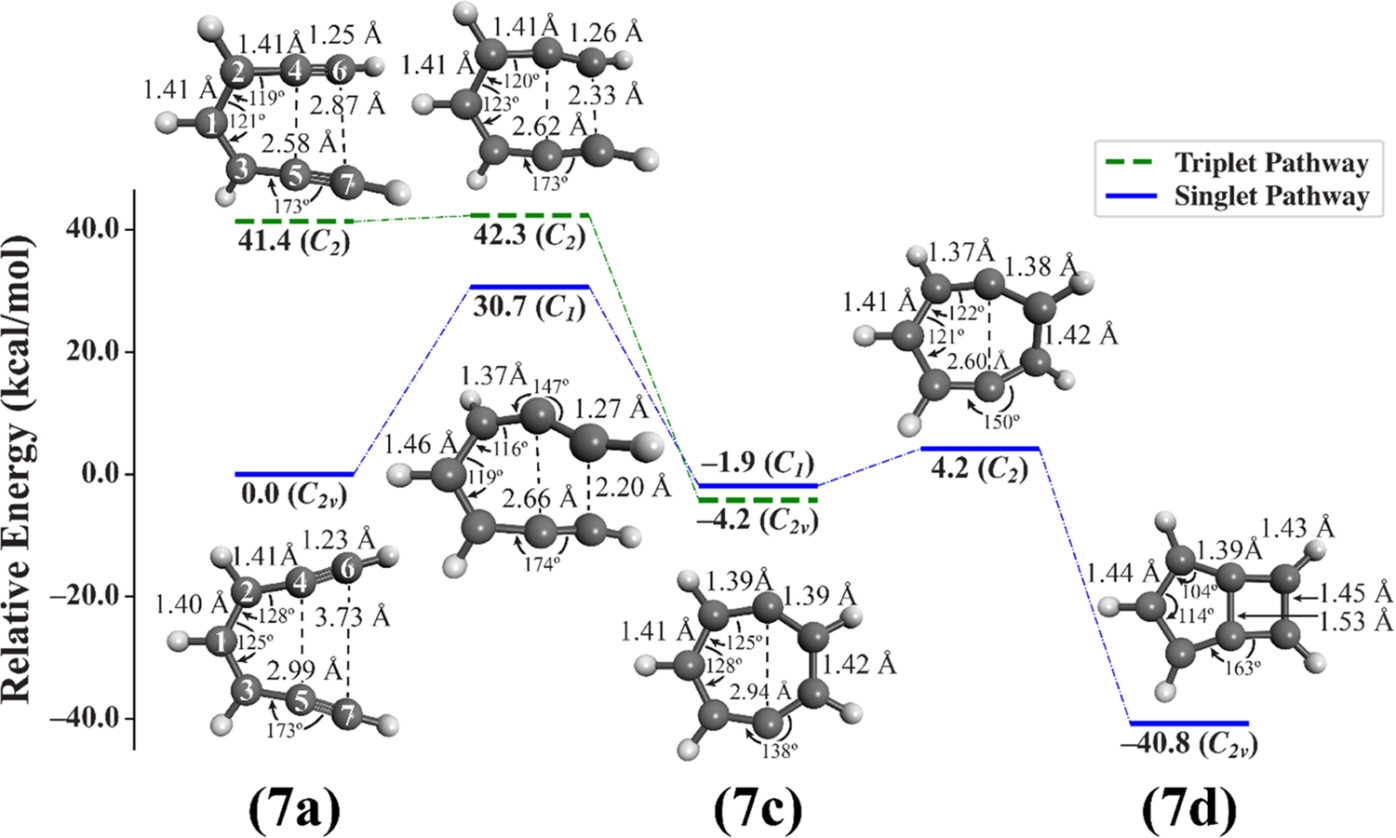
Reaction energy profile for singlet (solid blue lines) and triplet (dashed green lines) cyclization pathways of the hepta-1,6-diyne cation (Scheme 3, middle). Energies of all species were computed relative to the singlet reactant **7a** at the (SF-EOM)CCSD/cc-pVDZ level of theory, and geometries (structures inset) were optimized at these same levels of theory (for **7c**, the singlet geometry is shown). For convenience, we also provide geometric parameters for interatomic distances R(1,2) = R(1,3), R(2,4) = R(3,5), R(4,6) = R(5,7), R(4,5), and R(6,7) (Å) and bond angles ∠213, ∠421 = ∠135, and ∠357 = ∠246 (deg) for each optimized geometry; atom numbers are superimposed on the inset structure **7a**.

The reaction energy profile for the singlet cyclization of the hepta-1,6-diyne cation (blue solid lines) is shown in Figure 1 (atom numbering was overlaid upon the inset structure for **7a**). The reactant **7a** adopts a planar, *C*_*2*v_-symmetric structure in the *X*^1^*A*_1_ state, with slightly bent triple bonds (∠357 = ∠246 ≈ 173°). The equivalence of C_1_–C_2_ and C_1_–C_3_ bond lengths at ∼1.40 Å suggests that the positive charge of **7a** is well distributed among the central carbons C_3_–C_1_–C_2_. The geometry of **7a**, along with its *C*_2*v*_ symmetry, can be understood by examining the molecular orbitals (MOs) for this species, which are visualized in Figure S15a–c in the Supporting Information. The unrestricted highest-occupied molecular orbital (HOMO), lowest-unoccupied molecular orbital (LUMO), and LUMO + 1 in both α and β manifolds for the *X*^1^*A*_1_ state of the hepta-1,6-diyne cation (Figure S4b) resemble the idealized π molecular orbitals for the allyl system almost perfectly (Figure S4a), even though these orbitals are canonical (i.e., delocalized). Furthermore, the 2*b*_2_ orbital—resembling the idealized, totally bonding π_1_ orbital in the allyl system—is doubly occupied in this electronic state of **7a**, leading to both the C_3_–C_1_–C_2_ bond length equalization and this species’ planarity.

The distance between terminal acetylenic carbons C_6_···C_7_ of **7a** is ∼3.7 Å, larger than the critical range of approximately 2.9–3.4 Å previously hypothesized to be necessary for cyclization to occur in the 6-membered Bergman cyclization.^29,51,52^ Even so, the cyclization barrier along the singlet pathway is only +30.7 kcal mol^–1^, closely resembling the cyclization barrier of +30.4 kcal mol^–1^ computed by Luxon et al. for the canonical Bergman cyclization.^27^ These are relatively low barriers, considering that cyclization proceeds via a complicated, multiconfigurational, multistate singlet pathway. (For more details, see discussion in Section SIA in the Supporting Information for electronic configurations and states formed by symmetry-adapted linear combinations thereof in two-level systems representative of the diradicals examined here.)

Along the singlet pathway shown in Figure 1, the transition state (**7a** → **7c**) adopts a twisted, nonsymmetric (*C*_1_) structure, where the forming C_6_···C_7_ bond length is 2.2 Å. The formation of this bond generates **7c**, a two-configurational, closed-shell singlet diradical with a planar, *C*_1_ structure which is nearly of *C*_2*v*_ symmetry. Attempts to optimize the *C*_2*v*_**7c** structure were unsuccessful due to numerical instabilities present in the parallel version of the Q-Chem coupled cluster code, which can appear during geometry optimizations of structures with high symmetry.^63^ Regardless of the slightly nonsymmetric structure of the diradical product, this cyclization exhibits a total reaction energy of −1.9 kcal mol^–1^, compared to +5.8 kcal mol^–1^ computed by Luxon et al. at this level of theory for the canonical Bergman cyclization (value computed from those given in Table S3 in the Supporting Information of ref (27)). This increased stability of **7c** versus *p*-benzyne is likely due to decreased ring strain in this 7-membered cyclic diradical versus the 6-membered *p*-benzyne.

In addition to the singlet pathway to form **7c**, we have also identified a low-lying triplet pathway (Figure 1, dashed green bars with corresponding structures inset) which, once initiated from the 1 ^3^*B* state of triplet **7a** in *C*_2_ symmetry, proceeds through a transition state of the same symmetry to form a planar, *C*_2*v*_ structure of **7c** in the ^3^*B*_2_ state. Along this triplet surface, we optimized and frequency-confirmed all geometries using conventional coupled cluster theory with single and double substitutions (CCSD) where we access the high-spin triplet state directly rather than utilizing spin-flip excitations. For consistency with the EOM-SF-CCSD/cc-pVDZ treatment of species along the singlet pathway, these computations also utilized the same cc-pVDZ basis set and an unrestricted reference determinant.

Unlike the singlet ground state of reactant **7a**, the triplet state adopts a twisted, *C*_2_-symmetric structure. The triple bonds in this structure deviate from linearity by 7° to a bond angle of ∠357 = ∠246 ≈ 173°; however, unlike for the singlet structure, the triple bonds orient out of the plane to increase the dihedral angle between the acetylenic termini to D(6,2,3,7) = 31°, from the 25° for dihedral angle D(4,2,3,5). As with the *X*^1^*A*_1_ state of **7a** examined above, the geometric features of this twisted structure arise from the molecular orbitals occupied by this lowest-energy triplet state (1 ^3^*B*), visualized in Figure S4c in the Supporting Information. This triplet state of **7a** also exhibits three unrestricted molecular orbitals in both α and β manifolds resembling the idealized π system of the allyl cation; however, it singly occupies each of the 11*b* and 13*a* orbitals, which resemble the idealized totally bonding (π_1_) and nonbonding (π_2_) molecular orbitals of allene, respectively. By transferring an electron out of the totally π bonding 11*b* orbital into the π-nonbonding 13*a* orbital, the barrier to out-of-plane rotation is significantly reduced as compared to the *X*^1^*A*_1_ state of **7a**, thereby allowing for this lowest-energy triplet to adopt a twisted conformation that reduces the overall Coulombic repulsion between alkyne moieties. Furthermore, this twisting allows the alkynyl π systems to rotationally decouple from the π system of the central allyl cation moiety, thereby permitting the nonlinearity of the triple bonds to orient themselves in such a way as to increase the distance between -yne termini such that D(6,2,3,7) > D(4,2,3,5). Even with this exaggerated twist, however, the central bond angle ∠213 of 121° in the 1 ^3^*B* state of **7a** leads to a C_6_···C_7_ distance of only 2.87 Å, nearly a full Ångström closer than that in the singlet state.

Thanks to the decreased distance between the acetylenic termini in the triplet structure of **7a**, only minor geometric deformation is necessary to assume the structure of transition state **7a** → **7c** along the triplet pathway. Together with the ease of out-of-plane rotation facilitated by occupying the π-nonbonding molecular orbital of the central allyl cation moiety and reduced in-plane Coulombic repulsion between the alkyne termini, the triplet pathway has a low cyclization barrier of only +1 kcal mol^–1^. The structure for the triplet state of **7c**, while able to be optimized within *C*_2*v*_ symmetry, is geometrically similar to the singlet state with deviations of less than ±0.001 Å in C···C bond lengths and less than 0.2° in bond angles. The similarity in the molecular geometry of these states leads to a very small adiabatic singlet–triplet energy gap of +2.3 kcal mol^–1^, with the triplet being the ground state of **7c** at this geometry (*X*^3^*B*_2_). This stands in contrast to *p*-benzyne, whose ground state is a two-configurational, closed-shell singlet (TCS).^27^ We have verified the *X*^3^*B*_2_ ground state of **7c** by characterizing the energetic ordering of these states for **7c** at both singlet-optimized *C*_1_ and triplet-optimized *C*_2v_ geometries, provided in Table S1 in the Supporting Information, and determined that the *X*^3^*B*_2_ ground state of **7c** is insensitive to both symmetry considerations and minor geometric perturbations. A triplet ground state for **7c** may be due to a reduced through-bond coupling strength between radical centers in **7c** versus *p*-benzyne, as there is only one intervening, ideally oriented 1,4 σ bonding pathway^38,39^ connecting the radical centers of **7c** compared to two such connections in *p*-benzyne (for further details including the σ and σ* contributions to the overall wave function, see Section SIIC in the Supporting Information). Compared to the singlet state of **7a**, the formation of the triplet state of **7c** is only modestly exothermic, with a total cyclization energy of −4.2 kcal mol^–1^; however, this cyclization is extremely favorable on the triplet surface, with a total reaction energy of −45.6 kcal mol^–1^.

Interestingly, we have also determined that if **7c** is generated in the singlet state, a bond may form between the radical centers to form bicyclic species **7d**, as visualized in Figure 1. This symmetry-allowed intramolecular ring closure proceeds via a slightly twisted *C*_2_-symmetry transition state **7c** → **7d** whose minor geometric deformation affords another small activation barrier of +6.1 kcal mol^–1^. The resulting bicyclic product, **7d**, adopts a planar, *C*_2*v*_ structure, but without the bond length equalization present in the singlet and triplet structures of **7c**. Even with the *apparent* loss of aromaticity, however, **7d** is stabilized relative to **7c** by a significant −38.9 kcal mol^–1^, displaying the dominant thermodynamic advantage of forming σ bonds. Due to this significant thermodynamic stability and low barrier to ring closure, we hypothesize that if diradical **7c** were to be generated in its singlet state, it will be quickly and completely deactivated from being pharmacologically useful for H atom abstraction via the formation of **7d**.

**Scheme 5:**
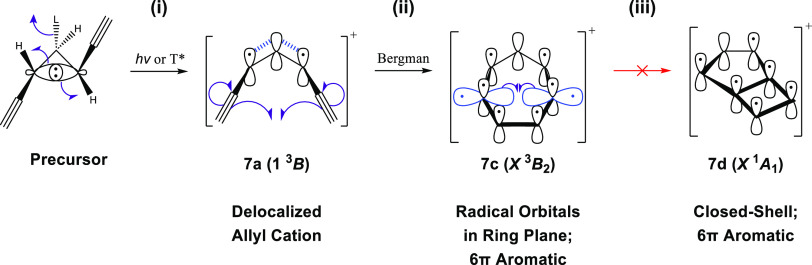
Proposed Orbital Mechanism for the Stepwise Cyclization of **7c** along the Triplet Pathway (i) Departure of a leaving group (L) and either photochemical excitation (*h*ν) or triplet sensitization (T*) of a cyclopropyl precursor leads to conrotatory ring opening to form reactant **7a** in its 1 ^3^*B* state; (ii) Bergman-like interrupted [2 + 2] cycloaddition of the alkyne termini of **7a** forms the cyclic diradical **7c** in its *X*^3^*B*_2_ state; (iii) further intramolecular radical elimination to form bicyclic **7d** in its *X*^3^*A*_1_ state is spin-forbidden.

The thermodynamic favorability of the formation of **7c** along the triplet pathway raises two questions: From where does this extraordinary stability originate? and Can this process be leveraged for pharmacological applications? Inspired by the generation of **7a** in the singlet state from a cyclopropyl precursor via a disrotatory ring opening, we propose that the triplet pathway for the formation of **7c** could be accessed via the conrotatory ring opening visualized in Scheme 5, which may be instantiated photochemically or via triplet sensitization.^64−66^ If **7a** could be generated in its triplet state, then the formation of diradical **7c** should proceed both quickly and completely, thanks to the combination of minimal barrier height and the high thermodynamic stability of **7c**. This diradical should be relatively long-lived not only due to its thermodynamic stability but also because the ring closure of the triplet state of **7c** to form **7d** is spin-forbidden. Triplet **7c** may also be accessed via an intersystem crossing from the singlet state. In the planar conformation of **7c**, the spin–orbit (SO) coupling should be zero, according to El-Sayed’s rule.^67^ However, under real conditions, the molecule flexes and bends such that the SO coupling may be intermittently enabled due to mixing between σ- and π-electrons (as in **7c** in Scheme 5).^68^ Although the barrier height separating singlet **7c** from **7d** is only +6.1 kcal mol^–1^, it may nevertheless lend **7c** a sufficiently long lifetime to be able to undergo intersystem crossing. Taken together, these observations lead us to believe that the formation of triplet **7c** may be experimentally relevant and worth further investigation (see ref (69) for a recent mini-review of photosensitizer agents and their action).

To understand why **7c** is so thermodynamically favorable, we have investigated the aromatic driving force for these cyclizations by performing isotropic NICS computations for singlet and triplet states of **7c** as well as for **7d**. These values are provided in Table 1, together with NICS analysis of benzene, *p*-benzyne, and both boat and chair conformers of cyclohexane for comparison. Based on the values for benzene and cyclohexane conformers, NICS values less than or equal to −8.0 indicate aromaticity, while those greater than or equal to −3.0 indicate nonaromaticity.

**Table 1:** NICS(−1, 0, +1) Indices for Cyclic Species **7c** and **7d**, as well as for *p*-Benzyne, Boat and Chair Cyclohexane, and Benzene

NICS index	boat^b^	chair^b^	benzene^b^	*p*-benzyne^a^	**7c** (singlet)^a^	**7c** (triplet)^c^	**7d** (cyclopentyl)^a^	**7d** (cyclobutyl)^a^
+1	–1.7	–2.0	–10.2	–19.0	–10.9	–11.1	–9.8	–11.6
0	–2.4	–2.0	–8.2	–28.6	–11.5	–10.8	–5.5	–1.2
–1	–3.1	–2.0	–10.2	–19.0	–10.9	–11.1	–9.8	–11.6

a BS-UB3LYP/6-311++G**.

b B3LYP/6-311++G**.

c UB3LYP/6-311++G**.

Using these qualitative ranges, both the singlet and triplet electronic states of **7c** are aromatic, consistent with the bond-length equalization in these species noted earlier. Surprisingly, however, both cyclopentyl and cyclobutyl ring moieties of **7d** appear to be aromatic despite the relative lack of bond-length equalization characteristic of typical aromatic species. The presence of aromaticity in the cyclobutyl moiety of **7d** is doubly surprising because cyclobutadiene—the monocyclic analogue to the four-membered ring moiety of **7d**—is known to be *anti*-aromatic! To investigate this further, we have computed isotropic deshielding values along an *xy*-scan connecting the two ring moieties’ geometric centers, raised 1 Å above the molecular plane (visualized in Figure S5 in the Supporting Information). These deshielding values (provided in Table S5 in the Supporting Information) demonstrate aromaticity along the entire scan, confirming that not only is the cyclobutyl moiety of **7d** aromatic but also the entire molecule is aromatic.

Where, then, does this aromaticity originate? And how does it evade not only the seeming lack of bond-length equalization but also what appears to be an antiaromatic ring in the cyclobutadiene moiety? Fortunately, some good old-fashioned electron pushing is sufficient to explain this behavior. Provided in Scheme 5 is an orbital-based exposition of the cyclization profiled in Figure 1, beginning with the formation of **7a** from its cyclopropyl precursor. After the departure of the leaving group from the precursor and conrotatory ring opening, there exist three unhybridized atomic 2*p*_*z*_ orbitals on carbons C_1_, C_2_, and C_3_, over which two electrons are distributed leading to a delocalized cation (**7a**). Next, the diradical species **7c** is formed by the interrupted [2 + 2] action of the Bergman cyclization, creating a π-network across the entire ring with 6 electrons occupying the seven contributing 2*p*_*z*_ orbitals. Therefore, **7c** conforms to the 4*n* + 2 rule, which conventionally defines aromaticity, so it should be no surprise that this species is so stable. Fortunately, the same picture also justifies the aromaticity observed in **7d**. Key to this is the realization that the radical electrons localized on carbons C_4_ & C_5_ occupy orbitals that are perpendicular to the π system, within the plane of the ring. Thus, the formation of the new C_4_–C_5_ bond in **7d** occurs without disturbing the out-of-plane 6π electrons that form the aromatic system. The source of the aromaticity in **7d** is therefore the delocalization of these π electrons around the perimeter of the bicyclic system rather than any interaction between anti/aromaticity present in the individual ring moieties of this species. From a structural point of view, **7d** can be thought of as a tropylium cation with a σ bond bisecting the seven-membered ring. The bridgehead σ bond (1.53 Å) is long because of the Mills–Nixon effect,^70^ which causes the four-membered ring in **7d** to resemble a dimethylene cyclobutene more so than a cyclobutadiene.

### Cyclization of the Octa-1,7-diyne Dication

Unlike for the cyclization of the hepta-1,6-diyne cation explored above, all attempts to optimize the hypothetical singlet diradical product **8c** proposed in Scheme 3 formed a closed bicyclic species **8d**, analogous to that of **7d**, which was stabilized relative to the singlet state of **8a** by −73.6 kcal mol^–1^ (Table S4) This is not surprising given the enhanced conformational flexibility of the 8-membered ring and the tremendous thermochemical driving force associated with the formation of the new C_5_···C_6_ σ bond. Unfortunately, we were unable to identify transition state **8a** → **8d**, which would define this concerted pathway on the singlet surface. However, we did profile this cyclization using the frozen-string approach^71^ at the UCCSD/cc-pVDZ level of theory on the lowest singlet surface, which provides an approximate upper bound on the activation barrier of this pathway of +41.8 kcal mol^–1^. While the sizable exothermicity of the double cyclization process would cause any **8a** present in the singlet state to thermodynamically convert to the pharmacologically inactive bicyclic species **8d**, the approximate +41.8 kcal mol^–1^ barrier to this process is sufficiently high that we expect that the direct thermal conversion of **8a**–**d** would be slow.

**Figure 2:**
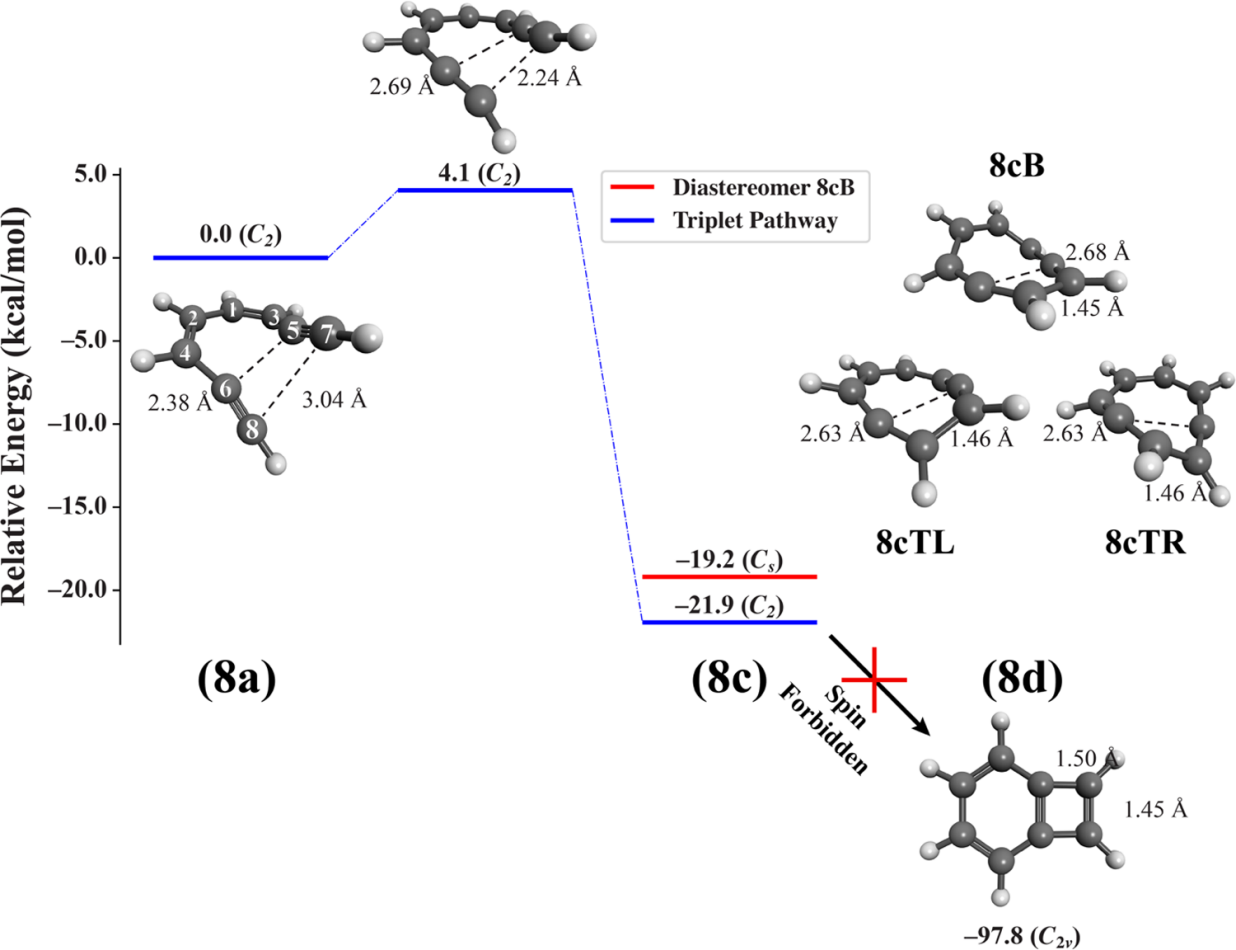
Reaction energy profile for the triplet pathway (solid blue lines) of the cyclization of the octa-1,7-diyne dication (Scheme 3, bottom). Energies of all species were computed relative to adiabatic triplet reactant **8a** at the UCCSD/cc-pVDZ level of theory, and geometries (structures inset) were optimized at these same levels of theory. For convenience, we provide geometric parameters for interatomic distances R(5,6) and R(7,8) (Å) for all species; atom numbering is superimposed over the inset structure for species **8a**.

We also identified a triplet cyclization pathway, visualized in Figure 2 (blue lines). Much like the triplet pathway of the hepta-1,6-diyne cation cyclization, the triplet state of reactant **8a** adopts a twisted, *C*_2_-symmetric structure, with acetylenic termini separated by ∼3.0 Å, a significantly smaller terminal distance than in the singlet geometry, where C_7_···C_8_ is separated by ∼4.7 Å. This C_7_···C_8_ distance decreases to ∼2.2 Å in the *C*_2_-symmetric transition state, with a barrier of only 4.1 kcal mol^–1^, before forming the diradical triplet **8c**.

Unlike **7c**, which adopted a single planar, *C*_2*v*_ structure on the triplet surface, we have confirmed the presence of three distinct conformations of the triplet product **8c** (structures inset in Figure 2): two inherently chiral, twisted conformations of *C*_2_ symmetry which we denote “twist-right” (**8cTR**) and “twist-left” (**8cTL**), as well as a boat-like conformer **8cB** of *C*_*s*_ symmetry. The geometric variety of **8c** is understandable given the increased conformational flexibility of the expanded 8-membered ring, and the inherent chirality of this molecule is due to the presence of two pseudoallene moieties surrounding each radical center within the ring. Indeed, **8cTL** and **8cTR** are nearly enantiomers of one another (geometric parameters agreeing to within ±0.1 Å), while **8cB** is a near-diastereomer of these. As expected, **8cTL** and **8cTR** are isoenergetic to within 0.1 kcal mol^–1^, while **8cB** is the least stable stereoisomer by nearly 3 kcal mol^–1^ (Figure 2, red line). It should be noted that the structures inset in Figure 2 demonstrate the formation of **8cTL**; however, the isoenergetic pathway forming **8cTR** simply proceeds through structures for **8a** and **8a** → **8c**, which are of inverted symmetry from those shown in Figure 2.

The activation barrier for the **8a** → **8c** cyclization of +4.1 kcal mol^–1^ is notably smaller than the +35.6 kcal mol^–1^ barrier on the triplet surface of the conventional Bergman cyclization^27^ and only slightly larger than the +1 kcal mol^–1^ barrier to the formation of **7c** on the triplet surface discussed above. A slightly higher cyclization barrier for the eight-membered system, relative to the seven-membered system, is likely due to Hammond’s postulate, supported by the fact that the C_7_···C_8_ distance in **8a** must shorten by only 0.8 Å in order to assume the transition state structure **8a** → **8c**, compared with the equivalent C_6_···C_7_ distance in **7a** needing to shorten by a mere 0.54 Å to assume the transition state structure of **7a** → **7c**. Interestingly, only transition states leading from left- and right-twisted conformations of **8a** to the formation of **8cTL** and **8cTR** could be identified, whereas **8cB** seems to not be able to form directly. Furthermore, no bona fide transition states could be identified for either **8cTL** → **8cB** or **8cB** → **8cTR** conformational transformations, making **8cB** seemingly inaccessible from the other minima on the triplet surface, despite its energy being only +2.7 kcal mol^–1^ higher than that of its twisted diastereomers.

Regardless of which twisted conformer of **8c** is formed, their stability relative to the reactant **8a**, together with the fact that a subsequent ring-closure process to form **8d** is spin-forbidden, leads to the conclusion that these are long-lived triplet diradicals. This stability could be due to the presence of aromaticity, as there are six π electrons to delocalize around the ring: two occupying the π orbitals of the butadiene dication moiety and another four left behind in the -ene units that remain after the Bergman cyclization. Unfortunately, the highly nonplanar and nonsymmetric nature of these species makes direct application of NICS to quantify the aromaticity in these systems unintuitive, as the locations for the probes used in NICS computations are typically defined relative to the ring plane, equidistant from all ring atoms. We have therefore generalized these conditions for placing NICS probes for symmetric, planar molecules to the nonsymmetric and nonplanar case, as discussed in Section SIC in the Supporting Information, and performed NICS computations to assess the presence of any aromaticity in the **8c** triplet conformers, along with singlet **8d**, in an identical manner to the computations described above for **7c** and **7d**. Presented in Table 2, NICS analysis indicates that both **8cTL** and **8cTR** are essentially aromatic, as all NICS indices for these twisted conformers hover around the qualitative cutoff value of −8.0 identified from the NICS indices of benzene presented in Table 1. On the other hand, the NICS indices for **8cB** much more closely resemble those for the boat conformer of cyclohexane (for both **8cB** and boat-cyclohexane, the +1 index is the one “riding” in the boat, whereas −1 is “below” the boat). This indicates that **8cB** is essentially nonaromatic, in agreement with the finding that this is the least stabilized conformer of **8c**. The closed-shell bicyclic species **8d** is also aromatic, and an analogous *xy*-scan above the plane of the molecule reveals that, much like **7d**, the aromaticity of **8d** arises from delocalization around the perimeter of the ring rather than any aromaticity localized to an individual cyclic moiety. This global aromaticity is, therefore, the cause of the extraordinary stability of **8d**.

**Table 2:** NICS(−1, 0, +1) Indices for Singlet **8d** and Each Conformer of Triplet **8c**

NICS index	**8cTL**^a^	**8cB**^a^	**8cTR**^a^	**8d** (cyclohexyl)^b^	**8d** (cyclobutyl)^b^
+1	–7.6	–2.3	–7.6	–7.9	–11.6
0	–8.3	–2.7	–8.3	–8.3	+3.0
–1	–7.6	–3.9	–7.6	–7.9	–11.6

a UB3LYP/6-311++G**.

b B3LYP/6-311++G**.

Similar to the formation of triplet **7c**, the aromatic stabilization of the twisted conformations of **8c** together with the low reaction barrier on the triplet surface makes this cyclization of possible interest for experimental study. While significant molecular design work would be needed to build the **8a** moiety into a biologically relevant scaffold in a synthetically accessible fashion, the vertical singlet–triplet gap for **8a** in its singlet geometry of +47.2 kcal mol^–1^ (Table S4), or 2.0 eV, suggests that it may be useful for photodynamic therapies. This singlet–triplet gap corresponds to an excitation wavelength of ∼606 nm. While a direct S0 → T1 transition is spin-forbidden, the nonplanarity of **8a** may allow for sufficient σ/π orbital mixing to produce spin–orbit coupling that could facilitate excitation to the triplet state. The penetration depth of visible light incident upon human skin increases with wavelength to a maximum of approximately 3.5 mm at λ = 1090 nm in the near-IR,^72^ before decreasing again for longer wavelengths due to the IR absorption of water^73^ (for a thorough review of light interactions with human tissue, see ref (74)). While not maximally penetrating, the 606 nm light required to vertically excite **8a** from its *X*^1^*A* state to the 1 ^3^*B* state falls within the so-called “therapeutic window” (see, e.g., Figure 3.6 in ref (74)), and indeed has been shown to penetrate up to approximately 1.5 mm of skin and 3 mm of mucosal tissue,^72^ making the triplet surface of **8a**—and therefore the cyclization forming long-lived triplet diradicals **8c**—of potential experimental interest.

### Comparing the Energetics for All Cyclization Reactions

Provided in Table 3 are barriers and reaction energies of all cyclization reactions discussed here together with the same data for the canonical Bergman cyclization of (*Z*)-hexa-3-ene-1,5-diyne. We find that the barriers to cyclization for these enediynes on their singlet surfaces decrease consistently with the size of the ring produced by the cyclization. The experimentally determined barrier height for the canonical Bergman cyclization of (*Z*)-hexa-3-ene-1,5-diyne of +28.8 kcal mol^–1^ slightly bucks this trend; however, the electronic barrier height of +34.3 kcal mol^–1^ computed by Luxon et al.^27^ at the same level of theory used in this work is consistent with the trend demonstrated here for our ionic cyclizations.

**Table 3:** Relative Energies for Barrier Heights and Total Reaction Energies for the Bergman-like Cyclizations Examined in This Study^a^

elementary step	pathway	Δ*E*^‡^ (kcal mol^–1^)	Δ_r_*E* (kcal mol^–1^)
Penta-1,4-diyne Anion			
**5a** → **5c**	OSS (*C*_*s*_)	+108.6	+106.5
	CSS (*C*_2*v*_)	+65.6	+32.9
	CSS (*C*_1_)		+21.8
(*Z*)-Hexa-3-ene-1,5-diyne			
theory^c^	CSS^b^	+34.3	+12.6
	triplet^c^	+32.9	–35.9
experiment^d^,^e^,^f^		+28.2^d^	+8.5^d^ or +13^e^
Hepta-1,6-diyne Cation			
**7a** → **7c**	singlet	+30.7	–1.9
	triplet	+0.96	–46.6
**7c** → **7d**	singlet	+6.1	–38.9
**7a** → **7d** (stepwise)	singlet	+30.7	–40.8
Octa-1,7-diyne Dication			
**8a** → **8cTL**	triplet	+4.1	–21.9
**8a** → **8d**	singlet	+41.8^f^	–73.9

a All barriers and reaction energies are adiabatic on their surfaces and were computed at the (SF-)UCCSD/cc-pVDZ level of theory for singlet and triplet pathways, respectively. Also included for comparison are theoretical and experimental estimates for the cyclization barrier and reaction energy of the canonical Bergman cyclization of (*Z*)-hexa-3-ene-1,5-diyne.

b Relative energies constructed from absolute electronic energies computed at the SF-CCSD/cc-pVDZ level of theory with an unrestricted reference wave function, taken from Table S3 and in the Supporting Information of ref (22).

c Relative energies constructed from absolute electronic energies computed at the CCSD/cc-pVDZ level of theory with an unrestricted reference wave function, taken from Table S7 and in the Supporting Information of ref (22).

d Value taken from Figure 4 in ref (5).

e Value taken from eq 10a in ref (75).

f Approximate upper bound to this cyclization barrier estimated along a frozen-string reaction pathway^71^ constructed along the lowest singlet surface and computed at the CCSD/cc-pDVZ level of theory using a restricted reference wave function.

In the larger systems (7- and 8-membered), the diradicals undergo a highly exothermic radical recombination to form a new σ bond. That recombination is hindered if the ring is small (i.e., *p*-benzyne); however, if the ring is larger, the recombination is easier, as **7d** and **8d** demonstrate.

Interestingly, the cyclization energies on the triplet surface for both the hepta-1,6-diyne cation and octa-1,7-diyne dication are both smaller than on their respective singlet surfaces—with the cyclization barrier of the hepta-1,6-diyne cation nearly nonexistent at only +1 kcal mol^–1^ due primarily to the fact that smaller geometric distortions are needed for each of the triplet reactants of these cyclizations to assume their transition state geometry than for their singlet counterparts.

A similar trend is observed for reaction energies on the singlet surface: with increasing ring size, the cyclization becomes progressively more thermodynamically favorable. Indeed, even though the cyclization energies for the penta-1,4-diyne anion and (*Z*)-hexa-3-end-1,5-diyne are endothermic, the singlet cyclization of the hepta-1,6-diyne cation is exothermic by −1.9 kcal mol^–1^. Unfortunately, the singlet cyclizations of both hepta-1,6-diyne cation and octa-1,7-diyne dication both lead to the formation of closed-shell bicyclic species, which, thanks to their global aromaticity, are so thermodynamically stable that we expect any formation of cyclic diradical products along these singlet pathways would be inactivated. On the other hand, the triplet cyclization pathways are both exothermic by −46.6 and −21.9 kcal mol^–1^ for the hepta-1,6-diyne cation and octa-1,7-diyne dication, respectively, thanks to the aromaticity present in their cyclic diradical products. Additionally, these triplet diradicals are spin-forbidden from further cyclizing to form the inactive bicyclic species, which we hypothesize will make these species long-lived and, therefore, potentially attractive for further study.

## Conclusions

We have characterized the energetics and aromatic driving force for Bergman-type cyclizations of ionic enediyne precursors, namely, the penta-1,4-diyne anion, the hepta-1,6-diyne cation, and the octa-1,7-diyne dication using high-level quantum chemical computations. While the 5-membered anionic system does indeed form a cyclized diradical, the energetic barrier to cyclization on the singlet surface is significantly large. We also see the formation of a diradical for the 7-membered cationic system, with a barrier to cyclization (∼31 kcal mol^–1^) comparable to the canonical 6-membered enediyne. We were unable to identify a diradical Bergman cyclization product for the octa-1,7-diyne dication on the singlet surface. On the triplet surfaces, both the hepta-1,6-diyne cation and octa-1,7-diyne dication enjoy small cyclization barriers of only +0.96 and +4.1 kcal mol^–1^, and exothermic reaction energies of −46.6 and −21.9 kcal mol^–1^, respectively. The exothermicity exhibited by these cationic Bergman cyclizations on the triplet surface is due to the presence of significant aromaticity, which we have quantified computationally via NICS analysis and justified with orbital arguments. Although we identified a pathway that would quench the diradicals of these 7- and 8-membered cyclizations on the singlet surface, the transformation of diradical to bicyclic σ bond formation is spin-forbidden when the diradicals are generated in their triplet states, making them both long-lived and thermodynamically stable.

## Methods

We performed geometry optimizations of all reactants, transition states, and products using Q-Chem 5.4.1. We utilized the spin-flip formulation of equation-of-motion coupled cluster theory with single and double substitutions (EOM-SF-CCSD)^53−62^ together with the correlation-consistent polarized-valence double-ζ (cc-pVDZ) basis set. Each optimized geometry was verified as a true minimum (for reactants and products) or first-order saddle point (for transition states) on the potential energy surface at this same level of theory via frequency analysis. All spin-flip computations leveraged an unrestricted, high-spin reference determinant; a thorough discussion of the theoretical background and practical advice for performing spin-flip coupled cluster computations is provided in Section SIA in the Supporting Information (see Associated Content for further details). While spin contamination can be an issue for unrestricted wave functions, and even more so for excited states based on unrestricted references, we have only observed minor spin contamination in our computations (see, e.g., ⟨*S*^2^⟩ values provided in Tables S2–S4 in the Supporting Information).

In addition to quantitatively characterizing the energetics of these cyclization processes, we also examined the aromaticity in the diradical products as a possible thermodynamic driving force for these reactions. Though taught in every introductory organic chemistry classroom, aromaticity is, itself, not easy to define.^76^ One metric we have chosen to use is NICS,^77^ which in its simplest formulation associates aromaticity with the negative value of the isotropic magnetic shielding computed at a point in space—either within or nearby a ring moiety in the molecule of interest—using a “probe atom” (i.e., an atom with no basis functions or nuclear charge). In its original incarnation, the NICS probe was placed at a central point within the ring, equidistant from all ring atoms,^77^ an approach now denoted NICS(0). Further probes may be placed 1 Å above and below the plane of the ring, similarly equidistant from all ring atoms, which are denoted NICS(+1) and NICS(−1), respectively. Taken together, these so-called “isotropic” NICS computations are simple, which has led to their popularity as an index of aromaticity, despite some well-known limitations.^78,79^

Due to their reduction of a molecule’s overall aromaticity to a single value computed for a particular point in space, these isotropic NICS indices are very sensitive to the choice of probe location. Unlike conventional aromatic molecules, which are both highly symmetric and perfectly planar, several of the cyclic species examined here are neither planar nor symmetric; therefore, there exist no unique points equidistant to all ring atoms, and the notions of “above” or below the ring are also ill-defined. To address this complication, we place the NICS(0) probe at the ring’s non-mass-weighted geometric centroid, and NICS(±1) probes 1 Å in either direction from that centroid along the ring’s principal moment of inertia, thereby uniquely defining reproducible probe locations (see Section SIB and Figure S2 in the Supporting Information for further details).

We have computed isotropic NICS values for all cyclic species in this work using the Gaussian16 program^80^ at the unrestricted, broken-symmetry BS-UB3LYP/6-311++G** level of theory, using the molecular geometries optimized at the EOM-SF-CCSD/cc-pVDZ level of theory using Q-Chem. This symmetry-breaking approach is necessary to describe the electron density of diradical singlets with reasonable accuracy, by mixing the highest-occupied and lowest-unoccupied Kohn–Sham orbitals.^81,82^

## Supporting Information Available

Zipped archive of molecular geometries for all stationary-point structures identified herein, each in plain-text XYZ format; additional theoretical background for diradical systems and the spin-flip approach; development of a general approach for placing NICS probes for nonsymmetric and nonplanar molecules; and additional tables, figures, and analysis (PDF)
