# Supplementary material for: Variations on the Bergman Cyclization Theme: Electrocyclizations of Ionic Penta-, Hepta-, and Octadiynes
Source: J Am Chem Soc. 2023 Sep 25;145(39):21408–18. doi: 10.1021/jacs.3c06691 (PMC10557144; doi:10.1021/jacs.3c06691)
Supplement: Supplementary file 1 — ja3c06691_si_001.pdf [file ja3c06691_si_001.pdf]

Supplementary Material to

Variations on the Bergman Cyclization Theme: Electrocyclizations of Ionic Penta-, Hepta-, and Octadiynes

Dominic A. Sirianni,[1] Xinli Song,[2] Salmika Wairegi,[2] Evan B. Wang,[2] Sebastian A. Mendoza-Gomez,[2] Adam Luxon,[2] Maxwell Zimmerly,[2] Ariana Nussdorf,[2] Michael Filatov(Gulak),[3] Roald Hoffmann,[4] and Carol A. Parish[2, a]

[1] *Department of Natural Sciences, Daemen University, Amherst, NY 14226*

[2] *Department of Chemistry, University of Richmond, Richmond, VA 23173*

[3] *Department of Chemistry, Kyungpook National University, Daegu 702-701, South Korea*

[4] *Department of Chemistry, Cornell University, Ithaca, NY 14853*

(Dated: 3 September 2023)

Warning: Don't ever print this document — it is long.

[a] Electronic mail: cparish@richmond.edu

Contents

S-I Theoretical Background **S-3**

A Electronic Structure Approaches for Diradical Molecules S-3

B Towards a General Approach for Placing NICS Probes S-5

S-II Supplementary Results & Discussion **S-8**

A Cyclization of the Penta-1,4-diyne Anion S-8

B Characterization of the S0 & T1 States of the Heptaen-1,6-diyne Cation (**7a**) S-11

C Characterization of the Electronic Structure of **7c** S-13

D Absolute & Relative Electronic Energies for All Cyclizations S-16

E Nucleus-Independent Chemical Shift Data S-20

List of Figures

S-1 Quantum chemistry on two-level systems S-4

S-2 Novel scheme for uniquely placing NICS probes S-6

S-3 Energetic Profile for Cyclization of the Penta-1,4-diyne Anion S-9

S-4 Allyl MOs for $X\ ^1A_1$ (S0) & $1\ ^3B$ (T1) States of **7a** S-11

S-5 Unrestricted molecular orbitals for $X\ ^3B_2$ State of **7c** in its C_1 geometry . . S-12

S-6 **8a**→**8d** FSM Profile S-19

S-7 NICS XY-Scan of **7d** & **8d** S-21

List of Tables

S-1 State energetics for **7c** S-14

S-2 Energetics for the cyclization of the penta-1,4-diyne anion S-16

S-3 Energetics for the cyclization of the heptaen-1,6-diyne cation S-17

S-4 Energetics for the cyclization of the octadien-1,7-diyne dication S-18

S-5 NICS Analyses of Cyclic Molecules S-20

S-6 NICS XY-Scan of **7d** & **8d** S-22

S-I. Theoretical Background

A. Electronic Structure Approaches for Diradical Molecules

We are interested in characterizing Bergman-like electrocyclization reactions, in which the reactants are closed-shell, ground state singlets and the products are (presumably) aromatic diradicals. Diradicals are theoretically challenging molecules[1,2] in which two electrons occupy two quasi-degenerate orbitals, the electronic states of which may be approximately described within the context of a two-level system, visualized schematically in Fig. S-1. For sufficiently small orbital energy splitting, the resulting six electronic configurations (Fig. S-1.a.i) combine to produce four symmetry-adapted electronic states: two closed-shell, two-configurational singlets (Ψ_{+}^{TCS} and Ψ_{-}^{TCS}), one open-shell singlet (Ψ^{OSS}), and three degenerate components of the triplet ($\Psi^{\mathrm{T}}_{M_s=-1}$, $\Psi^{\mathrm{T}}_{M_s=0}$, and $\Psi^{\mathrm{T}}_{M_s=+1}$), provided in Fig. S-1.a.ii-iii. Aside from the high-spin components of the triplet ($\Psi^{\mathrm{T}}_{M_s=\pm 1}$), each of these electronic states are multiconfigurational, requiring more than one Slater determinant to adequately describe the total wavefunction; the contribution to the total electronic energy of these additional determinants in the total wavefunction is typically referred to as *static electron correlation.*

While a relatively compact, two-configurational self-consistent field (TCSCF) wavefunction would be sufficient to capture the static correlation in this isolated two-level system, real diradicals are not so simple: in addition to the possibility of other quasidegenerate orbitals, whereby a more general, multiconfigurational self-consistent field (MCSCF) wavefunction is required, the contributions of instantaneous, pairwise electron-electron repulsion must also be captured. This dynamical electron correlation necessitates the use of a post-MCSCF, multireference approach, e.g., MR-PT, MR-CI, MR-CC, etc. in order to adequately describe the properties and chemical behavior of these molecules. Determining the reference active space (Fig. S-1.a.i) for molecules such as these is complicated, and MR approaches (Fig. S-1.a.ii-iii; e.g., MR-CI or MR-CC), are computationally intensive for even small systems with only a few heavy (i.e., non-hydrogen) atoms; for reviews of these theoretical approaches, see Refs 3 and 4, respectively. Furthermore, if the transition states in these Bergman-like cyclizations are product-like, they too may possess significant diradical character; due to the additional complications inherent to transition-state searches, the application of MR approaches to quantitatively examine diradical-producing reactions has also been limited.

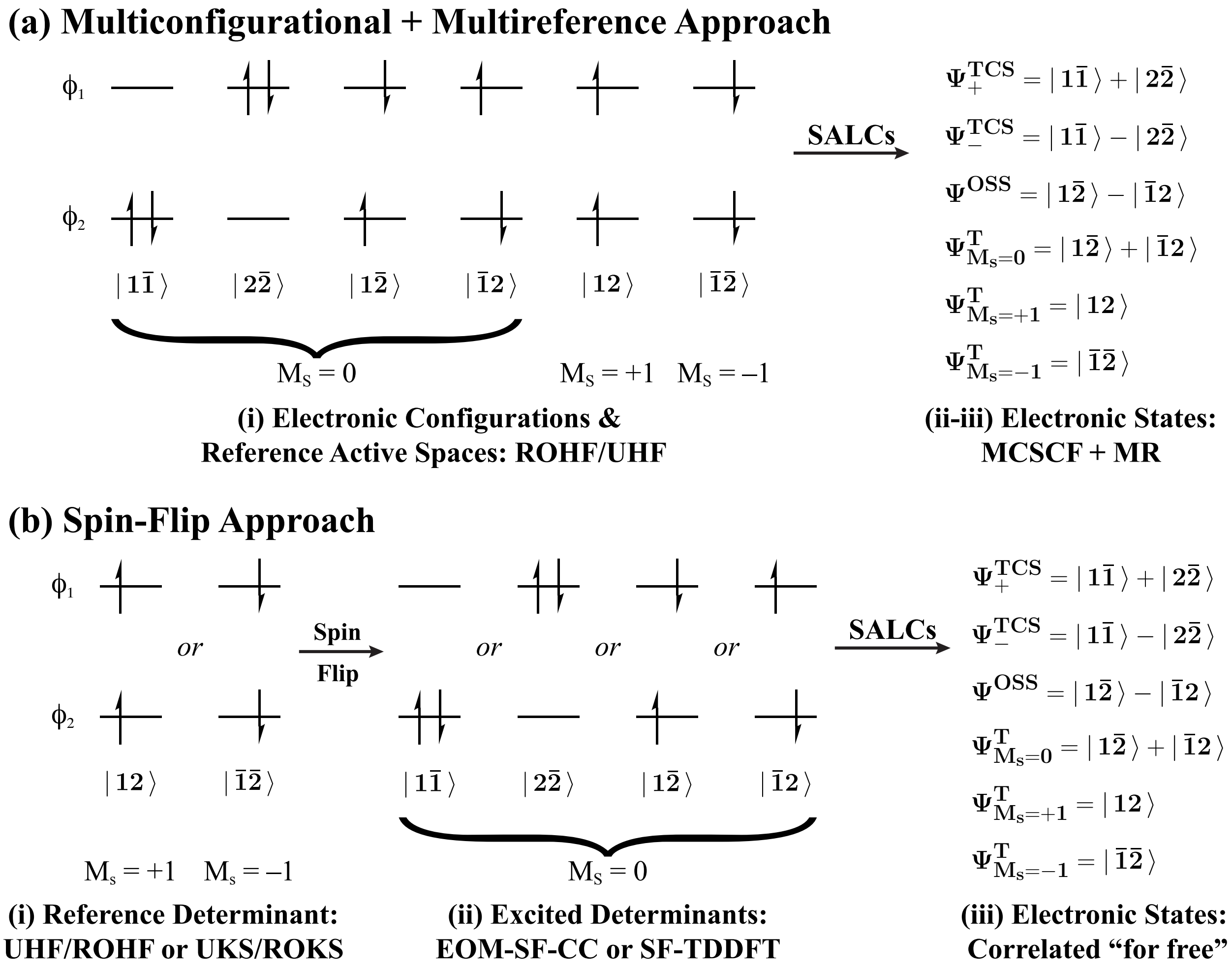

FIG. S-1. Schematic representations of two different approaches to study diradical molecules, within the context of a simple two-level system comprised of two electrons (labeled 1 and 2) in two quasidegenerate orbitals ϕ_1 and ϕ_2. Permutations of the orbital occupations in this two-level system produces six distinct electronic configurations, represented above as a Slater determinant $|\,\phi_1(i)\phi_2(j)\,\rangle \equiv |\,ij\,\rangle$ with electron i occupying the spatial orbital ϕ_1 and electron j occupying ϕ_2 and where an overbar denotes an electron has β spin.

Alternatively, spin-flip (SF) formulations of excited state methods based on single-reference theories like equation-of-motion coupled-cluster (SF-EOM-CC; abbreviated SF-CC)[5–9] or time-dependent density functional theory (SF-TDDFT)[10–13] have been developed that approach these systems in a different way, visualized in Fig. S-1.b. In the SF approach, a high-spin ($M_s = \pm 1$) triplet reference is constructed with an open-shell self-consistent field approach using either a restricted or unrestricted reference (UHF/ROHF or UK-S/ROKS; Fig. S-1.b.i), before spin-flip excitations being performed to generate all low-spin

($M_S = 0$) excited determinants (Fig. S-1.b.ii). These determinants may then be combined and symmetry-adapted using the squares of the transition amplitudes generating each spin-flipped determinant as expansion coefficients to generate multiconfigurational states for which both static and dynamical electron correlation is captured (Fig. S-1.b.iii). In this manner, the problem of applying a post-MCSCF multireference approach to capture both the static and dynamical electron correlation in these systems is reduced within the framework of a relatively more straightforward, computationally tractable single-reference theory where dynamical electron correlation is included "for free" (i.e., not requiring a subsequent correlated computation on top of the spin-flip treatment generating the multiconfigurational states of interest). Recently, the computational investigations of the canonical Bergman cyclization by Luxon *et al.* leveraging SF-CCSD have exhibited good agreement with experimental reaction barriers and thermodynamic quantities,[14] the success of which inspires us to apply this approach for the ionic extensions of this chemistry examined here.

B. Towards a General Approach for Placing NICS Probes

As discussed in the Methods section of the main text, the standard location for the placement of isotropic single-point NICS probes for planar, symmetric aromatic molecules is in the center of the ring plane [for NICS(0)] and 1 Å above and below the ring plane [for NICS($\pm$1)], all three of which must be equidistant from all ring atoms. For the highly nonsymmetric and nonplanar cyclic molecules examined here, however, there are two problems with this convention, namely (i) there does not exist a point (or points) which are mutually equidistant from all ring atoms and (ii) the notions of "above" and "below" are ill defined in the absence of a ring plane. While the first concern is easily circumvented by taking inspiration from the literature 3 where the NICS(0) probe location was originally defined to coincide with the non-mass–weighted geometric centroid of the ring, the second is still an open question with several conventions existing in the literature for symmetric, non-planar molecules.4 Rather than performing a much more involved and costly analysis based on mapping the isotropic NICS values along a grid surrounding our molecules, we have instead determined a system which is capable of unambiguously placing these probes in a manner analogous to NICS($\pm$1) for our nonsymmetric molecules that will recover previously reported conventions when applied also to symmetric molecules.

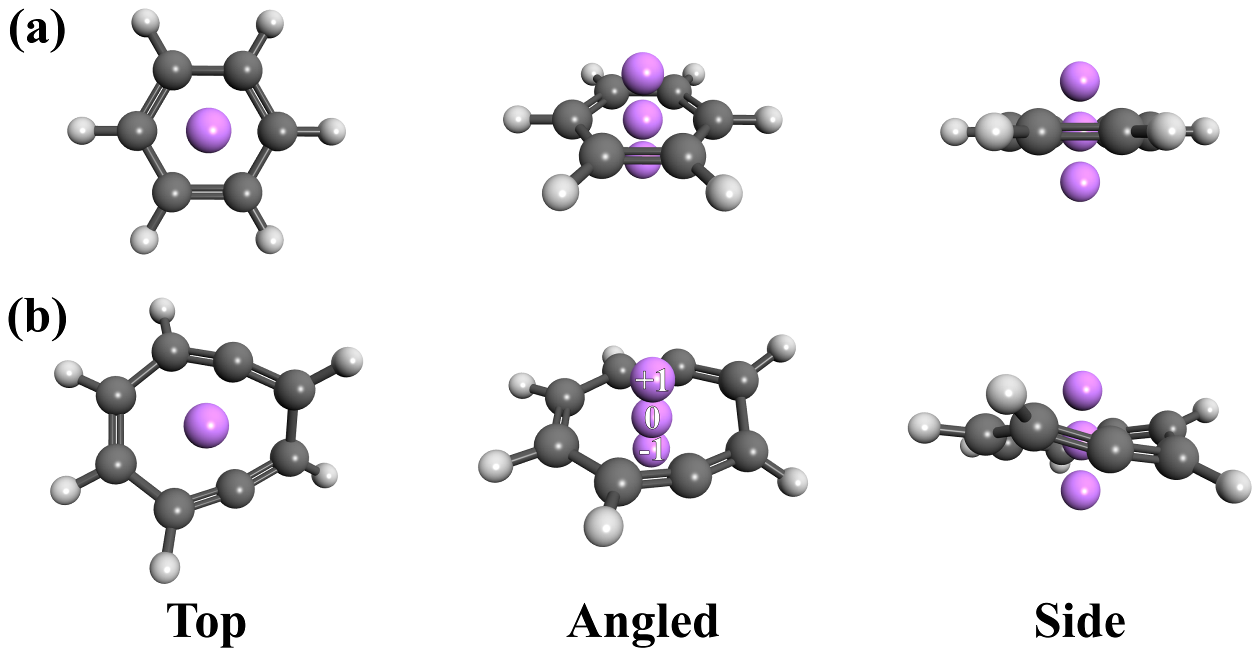

FIG. S-2. NICS(-1, 0, +1) probes (lilac spheres) superimposed on structures for (a) benzene and (b) **8cTL** as viewed from above (left panel), the side (right panel), or an angled perspective (center panel).

To derive where the NICS(±1) probes should be placed in the more general case of a non-symmetric, nonplanar molecule, it is intuitively sufficient to begin by examining where these probes are placed for the conceptually simplest aromatic molecule: benzene. In benzene, the NICS(0) probe is placed in the center of the ring plane, with NICS(±1) probes placed 1 Å perfectly above and below the molecular plane (see Fig. S-2.a for visualization). By connecting these points, it is clear that they lie on a line which coincides with the principal axis of rotation (i.e., the C_6 axis of the D_{6h} point group). While not all molecules possess a principal rotation axis defined by their symmetry, all molecules do possess a rotational reference frame defined by the rotational moments of inertia as originally outlined in the classic text by Wilson, Decius and Cross. 5 For symmetric molecules, the principal axis of rotation (by symmetry) does coincide with one of the vectors defining this rotational frame, namely the principal moment of inertia. These moments of inertia are defined to be the

eigenvectors of the moment of inertia tensor, $\underline{\underline{I}}$, a 3×3 matrix with elements given by

$$I_{\alpha\alpha} = \sum_n \left(\beta_n^2 + \gamma_n^2\right) \tag{1}$$

$$I_{\alpha\beta} = -\sum_n \alpha_n \beta_n \tag{2}$$

where α_n, β_n, and γ_n are mass-weighted Cartesian coordinates for atom n, within which the origin of the coordinate frame is defined to be the molecular center-of-mass. The three moments of inertia which define the rotational reference frame, $\{|\, i_n \,\rangle : n = a,\, b,\, c\}$, are the eigenvectors which diagonalize the moment of inertia tensor, $\underline{\underline{I}}$:

$$\underline{\underline{I}}|\, i_n \,\rangle = I_n|\, i_n \,\rangle \tag{3}$$

It is worth noting that the eigenvalues $\{I_n : n = a,\, b,\, c\}$ are related to the conventional (i.e., spectroscopic) rotational constants $N = A,\, B,\, C$ according to

$$N = \frac{h}{8\pi^2 I_n}. \tag{4}$$

To place the NICS($\pm$1) probes for a general, non-symmetric and non-planar molecule, we must take several additional considerations into account, namely

1. the atoms being considered in the construction of the moment of inertia tensor are only the ring atoms (i.e., the eight carbon atoms of 8cTL),
2. the origin of the coordinate system should be the non-mass–weighted centroid of the ring atoms, rather than the center-of-mass, and
3. we are only interested in the principal moment of inertia, which coincides with the principal symmetry axis for symmetric molecules.

Therefore, we may simply diagonalize the non-mass–weighted moment of inertia tensor $\tilde{\underline{\underline{I}}}$, constructed within the non-mass–weighted coordinate frame defined by coordinates $\tilde{\alpha}$, $\tilde{\beta}$, $\tilde{\gamma}$ and whose origin is placed at the ring centroid according to the eigenequation

$$\tilde{\underline{\underline{I}}}|\, \tilde{i}_n \,\rangle = \tilde{I}_n|\, \tilde{i}_n \,\rangle, \tag{5}$$

at which point our NICS($\pm$1) probes may be placed 1 Å in either direction along the principal moment of inertia in this reference frame, $|\, \tilde{i}_a \,\rangle$, as visualized in Fig. S-2.b for species

8cTL. For the convenience of the reader and as a service to the community, we have provided a script capable of automating this process written in the highly readable Python programming language. See `https://github.com/Parish-Lab/Bergman-Variations-578` for instructions to download, install, and use this script.

S-II. Supplementary Results & Discussion

A. Cyclization of the Penta-1,4-diyne Anion

The reaction energy profile for the cyclization of the penta-1,4-diyne anion is shown in Fig. S-3. Energetics for this cyclization were computed at the SF-EOM-CCSD/cc-pVDZ level of theory using SF-EOM-CCSD/cc-pVDZ optimized low-spin geometries (inset in Fig. S-3). For the reactant species **5a**, the C_1–C_2 and C_1–C_3 bonds are observed to be shorter than would be expected for a typical C–C single bond. Likewise, the acetylenic C_2–C_4 and C_3–C_5 bonds are longer than would be expected for a typical C–C triple bond. These bond compressions and elongations are likely due to π-electron donation from the formal π lone pair at C_1 into the π^* orbitals of the acetylene moieties. To put it another way, the perpendicular π system of this molecule is that of a pentadienyl anion, with attendant delocalization. Thanks to the symmetry specification required by the implementation of SF-CC in Q-Chem, both open-shell singlet (OSS) and two-component, closed-shell singlet (TCS) pathways could be found for this cyclization. Along the OSS pathway, the $C_4 \cdots C_5$ distance changes from 4.72 Å (reactant, **5a**) to 2.02 Å (transition state, **5a→5c**), before finally becoming 1.45 Å in the OSS product (**5c**). Both transition state **5a→5c** and product **5c** assume a nonplanar, C_s-symmetry structure reminiscent of the âĂIJenvelopeâĂİ conformer of cyclopentane, with central carbon C_1 puckered out of the ring plane by approximately 11° in **5a→5c** and 5° in **5c**.

While the $\angle C_3C_1C_2$ bond angle narrows from 123.2° in the reactant (**5a**) to 111.0° in the transition state (**5a→5c**), the OSS product **5c** actually exhibits a widening of this bond angle to 115°. This $\angle C_3C_1C_2$ widening in **5c** relative to **5a→5c** accompanies a lengthening of the distance between $C_2 \cdots C_3$ from 2.35 Å as the radical centers are forming in **5a→5c** to 2.41 Å between the radical centers in **5c**, a geometric change that reduces the Coulombic repulsion between the singly occupied radical orbitals of the OSS electronic state. With ac-

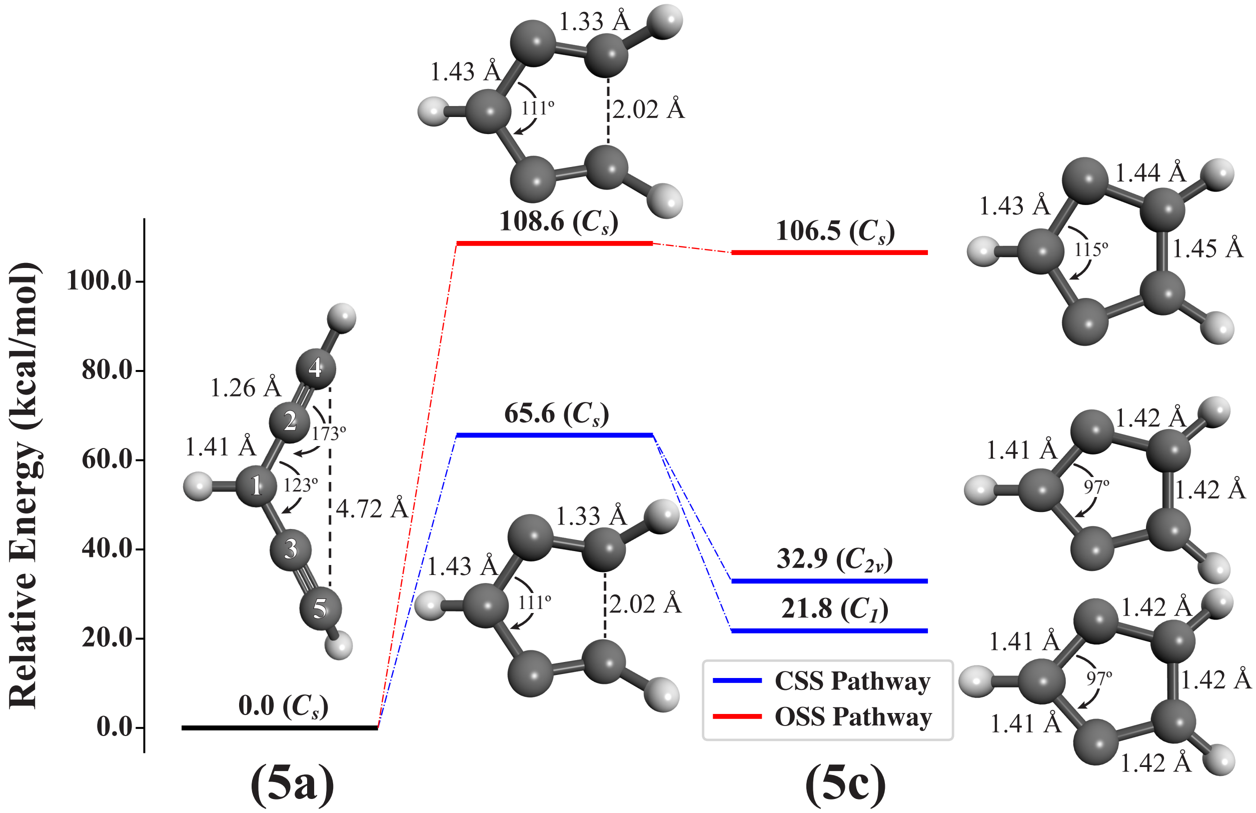

FIG. S-3. Reaction energy profile of open-shell singlet (red) and two-component closed-shell singlet (blue) pathways for the penta-1,4-diyne anion cyclization (Scheme 3, upper panel in the main text), computed at the SF-EOM-CCSD/cc-pVDZ level of theory for low-spin geometries of each species optimized at the same level of theory (inset). For convenience, we also provide the molecular point group symmetries and geometric parameters for interatomic distances R(1,2) = R(1,3), R(2,4) = R(3,5), and R(4,5) (Å), as well as bond angle ∠213 (°) for each optimized geometry, and ∠124 (°) for the reactant species.

tivation energy barriers for the OSS cyclization pathway of approximately +109 kcal mol^{-1} and a total reaction energy of approximately +107 kcal mol^{-1} (Fig. S-3), the cyclization of the penta-1,4-diyne anion is almost certainly too high to be observed *in vivo*. Earlier work on Bergman-type cyclizations of pentadiyne derivatives suggested that the rather high barrier and unfavorable total reaction energy arises from the formally forbidden [2+2]π character of the reaction during the initial stage of thermal ring closure.[15] Though this may be a contributing factor, it is more likely at least for the OSS surface that the unfavorable deformation energy required to bend the acetylene carbon atoms away from linearity

when adopting the transition state structure, together with the aforementioned unfavorable Coulombic repulsion between forming radical centers, conspire to play a larger role.

Along the TCS pathway for this cyclization, the transition state **5a**→**5c** assumes an identical structure to the OSS pathway, with the 43 kcal mol^{-1} difference in the energies of these two surfaces being entirely due to electronic effects. Alternatively, two markedly different structures were identified for the TCS state of **5c**: a planar, C_{2v} structure and a nonsymmetric C_1 structure, with bond lengths identical to its C_{2v} counterpart but whose ring carbons are nonplanar by only the slimmest of margins (dihedral angles of between only -0.06° and +0.07°). Other than the perfect or nearly perfect planarity for the TCS versus the markedly puckered OSS, the most significant structural difference between the geometries of these electronic states of **5c** is the distance between the radical centers $C_2 \cdots C_3$, which is 2.41 Å for the OSS versus only 2.12 Å in both C_{2v} and C_1 structures for the TCS. Not only are the radical centers significantly closer together in the TCS versus the OSS, but this pathway also boasts much less unfavorable cyclization energies of approximately $\Delta_r E = +22$ and $+33$ kcal mol^{-1} for the C_1 and C_{2v} structures, respectively.

Another marked difference between the OSS and TCS cyclization pathways is the energy gaps between the singlet state of interest (OSS or TCS) and the lowest triplet state. For the TCS pathway, the vertical singlet-triplet gap for the transition state **5a**→**5c** (i.e., the difference between singlet and triplet energies, both computed at the singlet geometry with the spin-flip procedure), $\Delta E_{\mathrm{ST}} = -15.2$ kcal mol^{-1}, as opposed to the OSS pathway with $\Delta E_{\mathrm{ST}} + 27.8$ kcal mol^{-1}. Furthermore, the cyclic product **5c** exhibits ΔE_{ST} = -19.5, -150.4, and +64.7 kcal mol^{-1} for the C_1 TCS, C_{2v} TCS, and C_s OSS, respectively. The exceptionally large ΔE_{ST} for the C_{2v} TCS versus the C_1 TCS showcases the reduced overlap of the singly-occupied triplet orbitals for the slightly nonplanar, C_1-symmetry TCS versus its planar C_{2v} counterpart, even though the intraradical $C_2 \cdots C_3$ distance is unchanged between these two structures. The triplet ground state observed for the nonplanar C_s structure of the OSS, with energy relative to reactant 5a of only +41.9 kcal mol^{-1} makes the triplet state of this conformer a potentially attractive synthetic target for use in anti-tumor applications. Taken together, these results show that the TCS pathway most closely resembles the canonical Bergman cyclization of (Z)-hexa-3-ene-1,5-diyne, which is not surprising as that cyclization proceeds via a TCS pathway.[14]

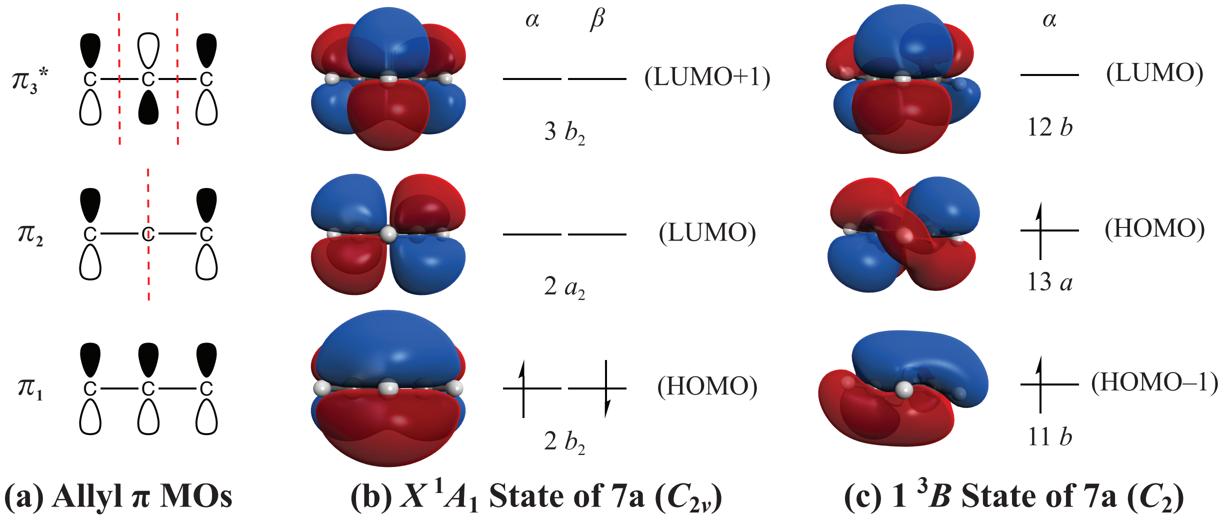

FIG. S-4. (a) π molecular orbitals of allene (adapted from Fig. 3.7 of Ref. 16); (b) unrestricted α & β MOs for the $X\ ^1A_1$ (S0) state of **7a** corresponding to the allyl π system; and (c) unrestricted α molecular orbitals for the 1 ^3B (T1) state of **7a**. All **7a** MOs are visualized "from behind," i.e., looking down the H–C_1 internuclear axis, and labeled by their orbital occupations and symmetries in the molecules' given point groups.

B. Characterization of the S0 & T1 States of the Heptaen-1,6-diyne Cation (7a)

The molecular geometries of the heptaen-1,6-diyne cation (**7a**) on both singlet and triplet surfaces display bond-length equalization in the central $C_3{-}C_1{-}C_2$ moiety, implying charge delocalization among these centers reminiscent of the allyl cation ($C_3H_3^+$). To examine this interpretation of the electronic structure of **7a**, we have computed molecular orbitals for both singlet ($X\ ^1A_1$, S0) and triplet (1 ^3B, T1) electronic states of **7a**, which are visualized in Fig. S-4.b,c together with the idealized π molecular orbitals of allene in Fig. S-4.a (adapted from from Fig. 3.7 of Ref. 16). Despite delocalization of the MOs for **7a** over the entire molecule, orbitals representative of the allyl π system were not challenging to identify, and closely parallel those expected for allene. Orbital occupations for these unrestricted MOs in both S0 and T1 states indicate that this moiety of **7a** is a two-electron system, confirming the resemblance identified above to the allyl cation and indicating that the cation of **7a** is indeed delocalized across this central allyl moiety.

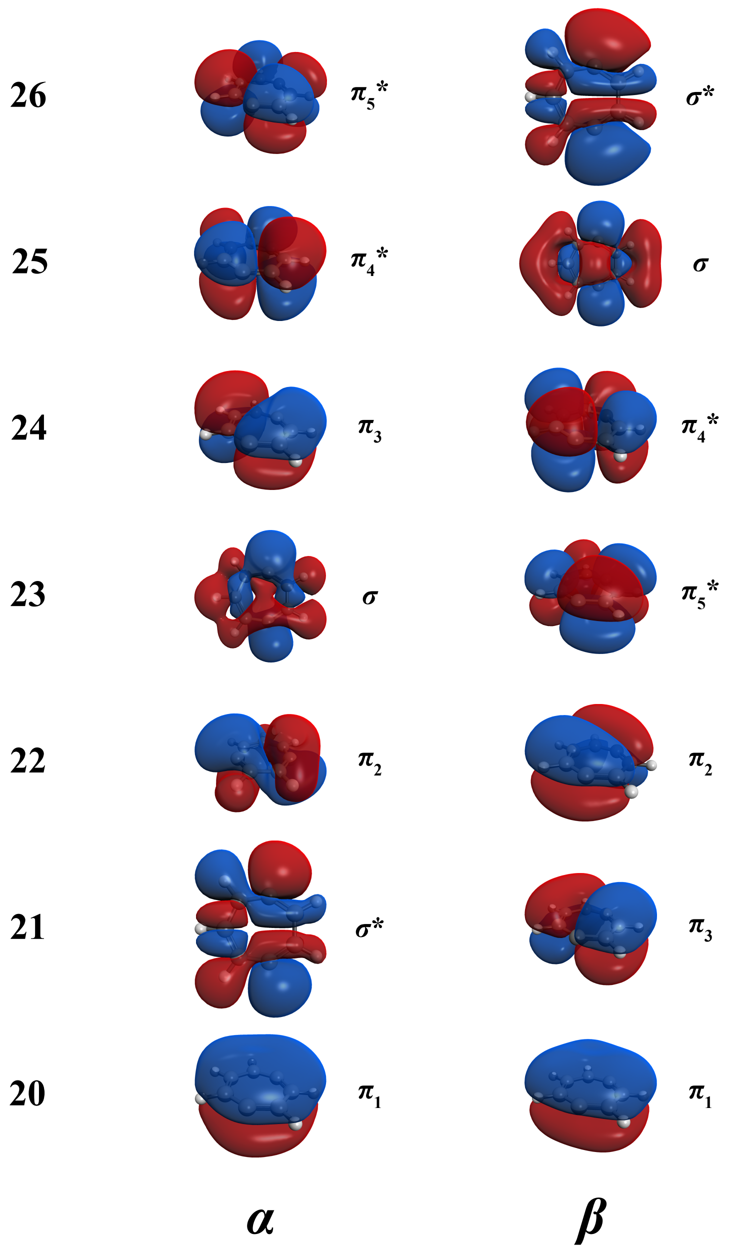

FIG. S-5. Unrestricted molecular orbitals for **7c** in its C_1 optimized geometry, computed at the UHF/cc-pVDZ level of theory, labeled according to their orbital numbers (in ascending energy order) and chemical designation. Chemical designation for orbitals in the β manifold are chosen to match the corresponding orbitals in the α manifold, i.e., the β_{21} orbital is labeled π_3 due to the orientation of its nodes matching that of π_3 in the α manifold (α_{24}).

C. Characterization of the Electronic Structure of 7c

While this work was under review, it was requested that we provide a slightly more detailed discussion of the electronic structure of **7c**, as there are multiple states in multiple geometries which must be considered holistically to understand the chemistry of this species. To this end, we have fully characterized the wavefunctions for the lowest high-spin triplet and lowest singlet states of **7c** at the singlet-optimized C_1 geometry. Provided in Fig. S-5 are unrestricted spin-up (α) and spin-down (β) molecular orbitals for **7c** at this geometry, computed for the high-spin triplet reference wavefunction at the UHF/cc-pVDZ level of theory, along with their orbital numbers and chemical "designation," i.e., their chemical interpretation in common parlance. We do note, however, that here "σ" and "σ^*" refer to the symmetric and antisymmetric combination, respectively, of the two quasidegenerate orbitals containing the radical electrons, oriented within the ring plane (perpendicular to the ring π system). The high-spin triplet wavefunction for **7c** at this geometry, denoted $|\, \Psi^{\mathrm{T}}_{M_s=+1} \rangle$ to be consistent with our earlier discussion in Section S-I A and Fig. S-1, has electronic configuration

$$|\, \Psi^{\mathrm{T}}_{M_s=+1} \rangle = |\, (\text{core})\ \ \pi_1\ \overline{\pi_1}\ \sigma^*\ \overline{\pi_3}\ \pi_2\ \overline{\pi_2}\ \sigma\ \pi_3 \rangle, \tag{6}$$

where orbital labels correspond to those orbitals visualized in Fig. S-5, overbars (e.g., $\overline{\pi_1}$) denote an unrestricted β orbital, and (core) represents the core electrons. This state can be more compactly represented by "pairing" the occupied molecular orbitals based on their chemical designation (and ordering them according to their relative energy in the α manifold):

$$|\, \Psi^{\mathrm{T}}_{M_s=+1} \rangle = |\, (\text{core})\, (\pi_1)^2\, (\sigma^*)^1\, (\pi_2)^2\, (\sigma)^1\, (\pi_3)^2 \rangle, \tag{7}$$

which clearly demonstrates that this 1^3B_2 triplet state singly occupies the two radical orbitals σ and σ^*, which is consistent with the lowest high-spin triplet state of p-benzyne.[14]

TABLE S-1. Total energies (kcal mol^{-1}) for indicated electronic states of **7c** at the given molecular geometry, relative to the energy of the heptaen-1,6-diyne cation (**7a**).

State[a]	Description[b]	C_1 [c]	C_{2v} [d]
$\vert \Psi^{\mathrm{T}}_{M_s=+1} \rangle$	Ref[e]	-4.05	-4.24
$\vert \Psi^{\mathrm{TCS}}_{+} \rangle$	TCS[f]	-1.87	-1.71
$\vert \Psi^{\mathrm{OSS}} \rangle$	OSS[f]	–	+110.93

[a] States named according to their generating excitation operator in the given point group; see text.

[b] Abbreviations describing electronic states. "TCS" = "two-component, closed-shell singlet," "OSS" = "open-shell singlet," "Ref" = "high-spin triplet reference."

[c] Energies computed at the C_1-symmetry geometry optimized at the EOM-SF-UCCSD/cc-pVDZ level of theory on the singlet surface for **7c**.

[d] Energies computed at the C_{2v}-symmetry geometry optimized at the UCCSD/cc-pVDZ level of theory on the triplet surface for **7c**.

[e] Energies of the reference determinant computed at the UCCSD/cc-pVDZ level of theory.

[f] Energies of all singlet excited states computed at the EOM-SF-UCCSD/cc-pVDZ level of theory.

As discussed above in Sec. S-I A, the spin-flip procedure works by applying a spin-flip excitation operator onto a high-spin triplet reference wavefunction. By closely examining the excitation amplitudes output from the spin-flip procedure, it is possible to construct the wavefunction for the lowest singlet state of **7c** at its singlet-optimized C_1 geometry:

$$| \Psi^{\mathrm{TCS}}_{+} \rangle = 0.41 \times | \, (\mathrm{core}) \; \pi_1 \, \overline{\pi_1} \, \sigma^* \, \overline{\pi_3} \, \pi_2 \, \overline{\pi_2} \, \pi_3 \, \overline{\sigma^*} \, \rangle + 0.32 \times | \, (\mathrm{core}) \; \pi_1 \, \overline{\pi_1} \; \overline{\pi_3} \, \pi_2 \, \overline{\pi_2} \, \sigma \, \pi_3 \, \bar{\sigma} \, \rangle + \ldots, \tag{8}$$

where we have included the two leading configurations in the multiconfigurational state (the next configuration only contributes to the total wavefunction with a weight of 0.027 = 2.7%), or again more compactly as

$$| \Psi^{\mathrm{TCS}}_{+} \rangle = 0.41 \times | \; (\mathrm{core}) \; (\pi_1)^2 \, (\sigma^*)^2 \, (\pi_2)^2 \, (\pi_3)^2 \, \rangle + 0.32 \times | \; (\mathrm{core}) \, (\pi_1)^2 \, (\pi_2)^2 \, (\sigma)^2 \, (\pi_3)^2 \, \rangle + \ldots \tag{9}$$

Based on its orbital occupations and the signs on its excitation amplitudes, this state clearly represents the constructive combination of the two-component closed-shell singlet $| \Psi^{\mathrm{TCS}}_{+} \rangle$ visualized in Fig. S-1.

Unlike *p*-benzyne, **7c** seems to have a high-spin triplet ground state — lower in energy than this two-component closed-shell singlet by 2.53 kcal mol^{-1}. This raises the question of whether the high-spin triplet is the ground state *only* at the singlet-optimized C_1 structure, or if it is the ground state also at the triplet-optimized C_{2v} structure. To address this possibility,

we have also computed at the EOM-SF-UCCSD/cc-pVDZ level of theory the total energies for the lowest two-configurational closed-shell singlet and open-shell singlet states of **7c** at this C_{2v} geometry, which are presented in Table S-1. Clearly, the electronic structure of **7c** is insensitive to the small geometric differences between the C_1 and C_{2v} structures, as both exhibit a high-spin triplet ground state. Furthermore, the lowest singlet state at each geometry is the two-configurational closed-shell singlet, and the singlet-triplet energy gaps between these lowest energy states are within 0.35 kcal mol^{-1} of each other. It seems, therefore, that this system does indeed have a high-spin triplet ground state, in contrast to the state ordering of *p*-benzyne,[14] which may indicate a reduction of through-bond coupling between the radical centers of **7c** relative to those of *p*-benzyne. As Hoffmann showed in 1968-'71,[17,18] sets of nonbonding electrons can interact both via spatial orbital overlap (termed "through-space coupling") and by coupling to the bonding/antibonding orbitals of an intervening σ bond (termed "through-bond coupling"). For *p*-benzyne, the two in-plane radical orbitals combine both symmetrically and antisymmetrically (see, e.g., Fig. 1 of Ref. 14); while through-space coupling of these orbitals would place the symmetric orbital (labeled "S" in Hoffmann's notation) lower in energy than the antisymmetric ("A") orbital, Hoffmann showed through purely symmetry-driven arguments that through-bond coupling reverses this order, such that A is lower in energy than S. For *p*-benzyne, the TCS ground state computed by Luxon *et al.* at the EOM-SF-UCCSD/cc-pVDZ level of theory is

$$| \Psi_+^{\mathrm{TCS}} \rangle = 0.53 \times | (\mathrm{A})^2 \rangle + 0.24 \times | (\mathrm{S})^2 \rangle, \qquad (10)$$

where the significantly larger weight of the $(\mathrm{A})^2$ configuration relative to the $(\mathrm{S})^2$ configuration — indicative of notable energetic splitting between the A and S orbitals — clearly demonstrates the presence of through-bond coupling in *p*-benzyne. It should be kept in mind that while the spin-flip approach optimizes orbitals of the single configurational, high-spin triplet reference, but not for the low-spin "excited states," it does allow for the description of the multiconfigurational nature of these low-spin states through the transition amplitudes of the spin-flip excitations, producing accurate orbital occupations with somewhat less than ideal molecular orbitals (i.e., only nonideal if the orbitals of the reference and excited states are significantly different shapes). Applying this same approach to analyze **7c**, the TCS computed at the same level of theory (but represented in Hoffmann's notation) is

$$| \Psi_+^{\mathrm{TCS}} \rangle = 0.41 \times | (\mathrm{A})^2 \rangle + 0.32 \times | (\mathrm{S})^2 \rangle, \qquad (11)$$

where the A and S radical orbitals correspond to σ^* and σ in Fig. S-5, respectively. The reduced weight of the $(A)^2$ configuration and increased weight of the $(S)^2$ configuration for the TCS of **7c**, relative to the ground state of *p*-benzyne, indicates that through-bond coupling is less significant in **7c**, in keeping with Hoffmann's original observation that trans 1,4 arrangement of radical lobes favors such coupling, of which **7c** only has one such pathway but two are present for *p*-benzyne.

D. Absolute & Relative Electronic Energies for All Cyclizations

TABLE S-2. Absolute (E_h) and relative (kcal mol^{-1}) energetics for all species in the cyclization of the penta-1,4-diyne anion (Scheme 3, top panel in the main text). Energies were computed at the indicated levels of theory at geometries optimized and frequency confirmed at this same level for the given electronic state within each species' point-group symmetry.

		High Spin		Low Spin			
**State**	**Point Group**	**Abs. Energy**	**Rel. Energy**	**Abs. Energy**	**Rel. Energy**	$\langle S^2 \rangle$	ΔE_{ST}[a]
			*penta-1,4-diyne anion, (**5a**)*[b]				
$X\ ^1A'$ (CSS)	C_s	–	–	−191.60428324559	0.00	0.00[c]	–
			*Transition State (**5a**→**5c**)*[d]				
$X\ ^1A'$ (CSS)	C_s	−191.4755058	80.81	−191.4997405	65.60	0.07[e]	15.21
$1\ ^1A''$ (OSS)	C_s	−191.4755058	80.81	−191.4312387	108.59	0.53[e]	−27.78
			*2,3-didehydrocyclopentene anion, (**5c**)*[d]				
$X\ ^1A$ (CSS)	C_1	−191.5385353	41.26	−191.5696083	21.76	0.02[e]	19.50
$1\ ^1A_1$ (CSS)	C_{2v}	−191.3121352	183.33	−191.5518475	32.91	0.04[e]	150.42
$1\ ^1A'$ (OSS)	C_s	−191.5375436	41.88	−191.4345019	106.54	0.74[e]	−64.66

[a] Singlet–triplet gap taken to be the energy of transition from singlet to triplet surface.

[b] Energy computed at the CCSD/cc-pVDZ level of theory with a restricted, closed-shell reference determinant

[c] $\langle S^2 \rangle$ values computed for Hartree–Fock reference determinant

[d] Energies computed at the EOM-SF-CCSD/cc-pVDZ level of theory with an unrestricted, high-spin open-shell reference determinant

[e] $\langle S^2 \rangle$ values computed for low-spin excited states of EOM-SF-CCSD procedure

TABLE S-3. Absolute (E_h) and relative (kcal mol^{-1}) energetics for all species in the singlet and adiabatic triplet cyclization pathways of the heptaen-1,6-diyne cation (Scheme 3, middle panel in the main text). Energies were computed at the indicated levels of theory at geometries optimized and frequency confirmed at this same level for the lowest-energy singlet (top) and adiabatic triplet (bottom) surfaces within each given point-group symmetry.

				High Spin		Low Spin			
**Species**	**State**		**Point Group**	**Absolute Energy**	**Relative Energy**	**Absolute Energy**	**Relative Energy**	$\langle S^2 \rangle$	ΔE_{ST}[a]
				Singlet Surface					
**7a**[b]	$X\ ^1A_1$	(CSS)	C_{2v}	–	–	−268.482101498714	0.00	0.00[d]	–
**7a→7c**[c]	$X\ ^1A$	(CSS)	C_1	−268.3853812	60.69	−268.433257	30.65	0.10[e]	30.04
**7c**[c]	$1\ ^1A$	(CSS)	C_1	−268.4885569	−4.05	−268.4850775	−1.87	0.09[e]	−2.18
**7c→7d**[c]	$X\ ^1A$	(CSS)	C_2	−268.474087	5.03	−268.4754073	4.20	0.11[e]	0.83
**7d**[b]	$X\ ^1A_1$	(CSS)	C_{2v}	–	–	−268.5471103	−40.79	0.00[d]	–
				Adiabatic Triplet Surface[f,g]					
**7a**	$1\ ^3B$	(T0)	C_2	−268.416188005966	41.36	–	–	2.59[d]	41.36
**7a→7c**	$1\ ^3B$	(T0)	C_2	−268.4146552	42.32	–	–	2.49[d]	11.67
**7c**	$X\ ^3B_2$	(T0)	C_{2v}	−268.4888543	−4.24	–	–	2.60[d]	−2.37

[a] Singlet–triplet gap taken to be the energy of transition from singlet to triplet surface.

[b] Energies computed at the CCSD/cc-pVDZ level of theory with a restricted, closed-shell reference determinant

[c] Energies computed at the EOM-SF-CCSD/cc-pVDZ level of theory with an unrestricted, high-spin open-shell reference determinant

[d] $\langle S^2 \rangle$ values computed for Hartree–Fock reference determinant

[e] $\langle S^2 \rangle$ values computed for low-spin excited states of EOM-SF-CCSD procedure

[f] Energies computed at the UCCSD/cc-pVDZ level of theory with an unrestricted, high-spin open-shell reference determinant

[g] Relative energies computed with respect to $X\ ^1A_1$ state of **7a**

TABLE S-4. Absolute (E_h) and relative (kcal mol^{-1}) energetics for all species in the cyclization of the octadien-1,7-diyne dication (Scheme 3, bottom panel in the main text). Energies were computed at the indicated levels of theory at geometries optimized and frequency confirmed at this same level for the lowest-energy singlet (top) and adiabatic triplet (bottom) surfaces within each given point-group symmetry.

**Species**	**State**		**Point Group**	**Absolute Energy**	**Relative Energy**	$\langle S^2 \rangle^{\mathbf{a}}$	$\Delta E_{ST}{}^{\mathbf{b}}$
			Singlet Surface[c]				
**8a**	X^1A	(CSS)	C_2	−306.5739104	0.00	0.00	47.16[d]
**8d**	X^1A_1	(CSS)	C_{2v}	−306.6916025	−73.85	0.00	–
			Adiabatic Triplet Surface[e]				
**8a(L)**	1^3B	(T0)	C_2	−306.5357421	23.95	2.39	23.95[f]
**8a(L)→8cTL**	1^3B	(T0)	C_2	−306.529268	28.01	2.50	–
**8cTL**	1^3B	(T0)	C_2	−306.5706965	2.02	2.84	–
**8a(R)**	1^3B	(T0)	C_2	−306.5357421	23.95	2.39	23.95[f]
**8a(R)→8cTR**	1^3B	(T0)	C_2	−306.529268	28.01	2.50	–
**8cTR**	1^3B	(T0)	C_2	−306.5706966	2.02	2.84	–
**8cB**	$1^3A''$	(T0)	C_s	−306.5663204	4.76	2.87	–

[a] $\langle S^2 \rangle$ values computed for Hartree–Fock reference determinant

[b] Singlet–triplet gap taken to be the energy of transition from singlet to triplet surface.

[c] Energies computed at the CCSD/cc-pVDZ level of theory with a restricted, closed-shell reference determinant

[d] Vertical singlet-triplet energy gap

[e] Energies computed at the UCCSD/cc-pVDZ level of theory with an unrestricted, high-spin open-shell reference determinant

[f] Adiabatic singlet-triplet energy gaps

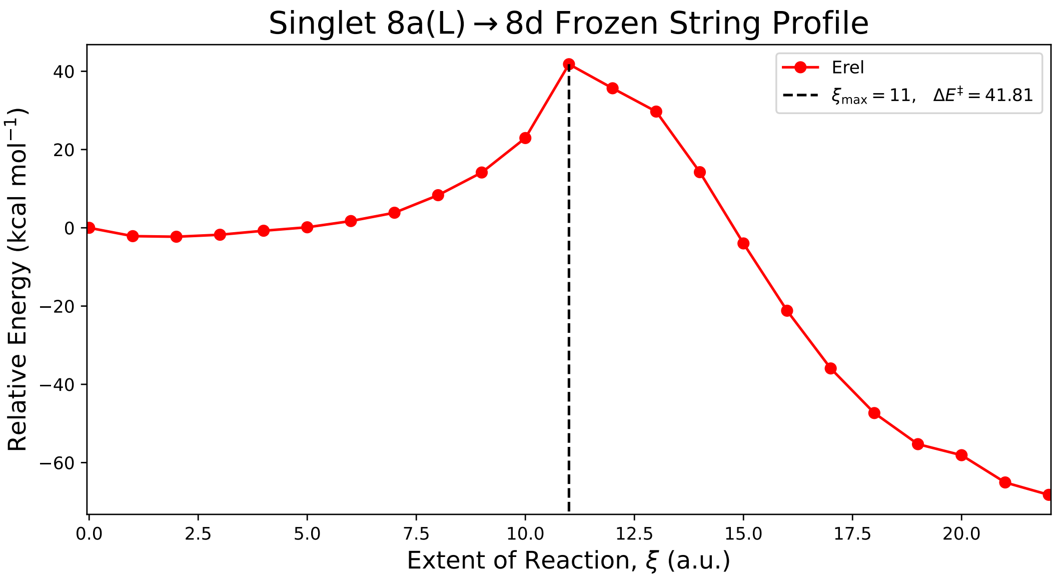

FIG. S-6. Relative electronic energies (kcal mol^{-1}) computed along the frozen-string pathway connecting the left-twisted conformation of the singlet structure of **8a** to the bicyclic species **8d**, computed at the UCCSD/cc-pVDZ level of theory with an unrestricted reference determinant.

E. Nucleus-Independent Chemical Shift Data

TABLE S-5. Nucleus-independent chemical shifts (NICS) for all cyclic species considered here, computed using Gaussian09 at the indicated level of theory.

Species	State	Point Group	NICS(−1)	NICS(0)	NICS(+1)
Singlet cyclization of the penta-1,4-diyne anion[a]					
**5c**	$X\ ^1A$ (CSS)	C_1	−4.0026	−0.7977	−4.0009
**5c**	$1\ ^1A_1$ (CSS)	C_{2v}	−3.9966	−0.7936	−3.9966
**5c**	$1\ ^1A'$ (OSS)	C_s	−0.5176	−6.1412	5.6688
Singlet cyclization of the heptaen-1,6-diyne cation					
**7c**[a]	$1\ ^1A$ (CSS)	C_1	−10.9432	−11.5370	−10.9432
**7d (cyclopentyl)**[b]	$X\ ^1A_1$ (CSS)	C_{2v}	−9.7586	−5.4856	−9.7586
**7d (cyclobutyl)**[b]	$X\ ^1A_1$ (CSS)	C_{2v}	−11.5955	−1.1566	−11.5955
Triplet cyclization of the heptaen-1,6-diyne cation[c]					
**7c**	$X\ ^3B_2$ (T0)	C_{2v}	−11.0919	−10.8340	−11.0919
Singlet surface of the octadien-1,7-diyne dication					
**8d (cyclohexyl)**[b]	$X\ ^1A_1$ (CSS)	C_{2v}	−7.9092	−3.0547	−7.9092
**8d (cyclobutyl)**[b]	$X\ ^1A_1$ (CSS)	C_{2v}	−11.6330	3.0410	−11.6327
Triplet cyclization of the octadien-1,7-diyne dication[c]					
**8cTL**	$1\ ^3B$ (T0)	C_2	−7.5583	−8.3285	−7.5583
**8cTR**	$1\ ^3B$ (T0)	C_2	−7.5622	−8.3319	−7.5622
**8cB**	$1\ ^3A''$ (T0)	C_s	−3.8493	−2.6983	−2.2972
Reference molecules for aromaticity/non-aromaticity[b]					
**Benzene**	$X\ ^1A_{1g}$ (CSS)	D_{6h}	−10.1892	−8.1533	−10.1891
**Cyclohexane (boat)**[d]	$X\ ^1A_1$ (CSS)	C_{2v}	−3.0504	−2.4445	−1.7285
**Cyclohexane (chair)**	$X\ ^1A_{1g}$ (CSS)	D_{3d}	−1.9538	−1.9957	−1.9538

[a] NICS computed at the BS-UB3LYP/6-311++G** level of theory with an unrestricted, low-spin open-shell reference determinant and utilizing 50% symmetry-breaking

[b] NICS computed at the B3LYP/6-311++G** level of theory with a restricted, closed-shell reference determinant

[c] NICS computed at the UB3LYP/6-311++G** level of theory with an unrestricted, high-spin open-shell reference determinant

[d] the NICS(+1) probe is "riding in the boat," while the NICS(−1) probe is "below the boat"

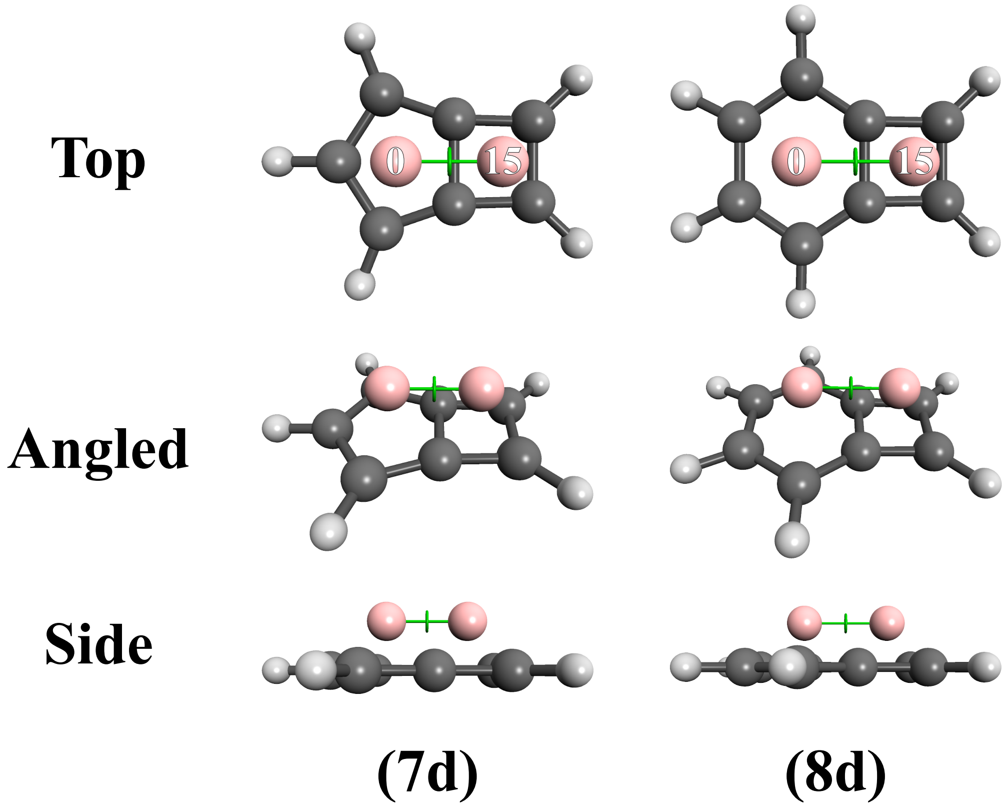

FIG. S-7. NICS probe locations for the *xy*-scans between the NICS(+1) probes above the two ring moieties in each of **7d** and **8d**; all probes along the scan coordinate remain +1 Å above the molecular plane.

TABLE S-6. Nucleus-independent chemical shifts (NICS) along an xy scan between the NICS(+1) probes above the two ring moieties of **7d** and **8d**, remaining +1 Å above the molecular plane along the scan, computed using Gaussian09 at the B3LYP/6-311++G** level of theory with a closed-shell, restricted reference determinant.

Probe Identity	7d	8d
**0**[a]	−9.7586	−7.9092
**1**	−5.4856	−3.0547
**2**	−9.7586	−7.9092
**3**	−11.5955	−11.6327
**4**	−1.1566	3.0410
**5**	−11.5955	−11.6327
**6**	−9.7586	−7.9092
**7**	−9.5778	−7.8025
**8**	−10.0028	−8.3456
**9**	−10.8739	−9.5119
**10**	−11.8842	−11.0836
**11**	−12.6525	−12.5840
**12**	−12.8913	−13.4376
**13**	−12.5825	−13.3576
**14**	−12.0041	−12.6131
**15**[b]	−11.5688	−11.8494

[a] Initial NICS probe centered above the cyclopentyl and cyclohexyl ring moieties of **7d** and **8d**, respectively.

[b] Final NICS probe centered above the cyclobutyl ring moieties of **7d** and **8d**.

References

[1] W. T. Borden, in *ACS Symposium Series*, Vol. 1209, edited by E. T. Strom and V. V. Mainz (American Chemical Society, Washington, DC, 2015) pp. 251–303.

[2] T. Stuyver, B. Chen, T. Zeng, P. Geerlings, F. De Proft, and R. Hoffmann, Chemical Reviews **119**, 11291 (2019).

[3] P. G. Szalay, T. Müller, G. Gidofalvi, H. Lischka, and R. Shepard, Chem. Rev. **112**, 108 (2012).

[4] D. I. Lyakh, M. Musia\l, V. F. Lotrich, and R. J. Bartlett, Chem. Rev. **112**, 182 (2012).

[5] P. U. Manohar and A. I. Krylov, The Journal of Chemical Physics **129**, 194105 (2008).

[6] S. V. Levchenko and A. I. Krylov, The Journal of Chemical Physics **120**, 175 (2004).

[7] A. I. Krylov, L. V. Slipchenko, and S. V. Levchenko, in *Electron Correlation Methodology*, ACS Symposium Series, Vol. 958 (American Chemical Society, 2007) Chap. 6, pp. 89–102.

[8] A. I. Krylov and C. D. Sherrill, The Journal of Chemical Physics **116**, 3194 (2002).

[9] A. I. Krylov, Chemical Physics Letters **338**, 375 (2001).

[10] Y. A. Bernard, Y. Shao, and A. I. Krylov, The Journal of Chemical Physics **136**, 204103 (2012).

[11] D. Casanova and M. Head-Gordon, The Journal of Chemical Physics **129**, 064104 (2008).

[12] S. Lee, M. Filatov, S. Lee, and C. H. Choi, The Journal of Chemical Physics **149**, 104101 (2018).

[13] X. Zhang and J. M. Herbert, The Journal of Chemical Physics **143**, 234107 (2015).

[14] A. R. Luxon, N. Orms, R. Kanters, A. I. Krylov, and C. A. Parish, The Journal of Physical Chemistry A **122**, 420 (2018).

[15] S. P. Kawatkar and P. R. Schreiner, Organic Letters **4**, 3643 (2002).

[16] I. Fleming, *Molecular Orbitals and Organic Chemical Reactions*, 1st ed. (Wiley, 2010).

[17] R. Hoffmann, A. Imamura, and W. J. Hehre, Journal of the American Chemical Society **90**, 1499 (1968).

[18] R. Hoffmann, Accounts of Chemical Research **4**, 1 (1971).
